# Supplementary material for: Phytochemical Profiling, Antioxidant and Cognitive-Enhancing Effect of Helichrysum italicum ssp. italicum (Roth) G. Don (Asteraceae)
Source: Plants (Basel). 2023 Jul 25;12(15):2755. doi: 10.3390/plants12152755 (PMC10420817; doi:10.3390/plants12152755)
Supplement: Supplementary file 1 [file plants-12-02755-s001.zip › plants-2517813-supplementary.pdf]

Supplementary material

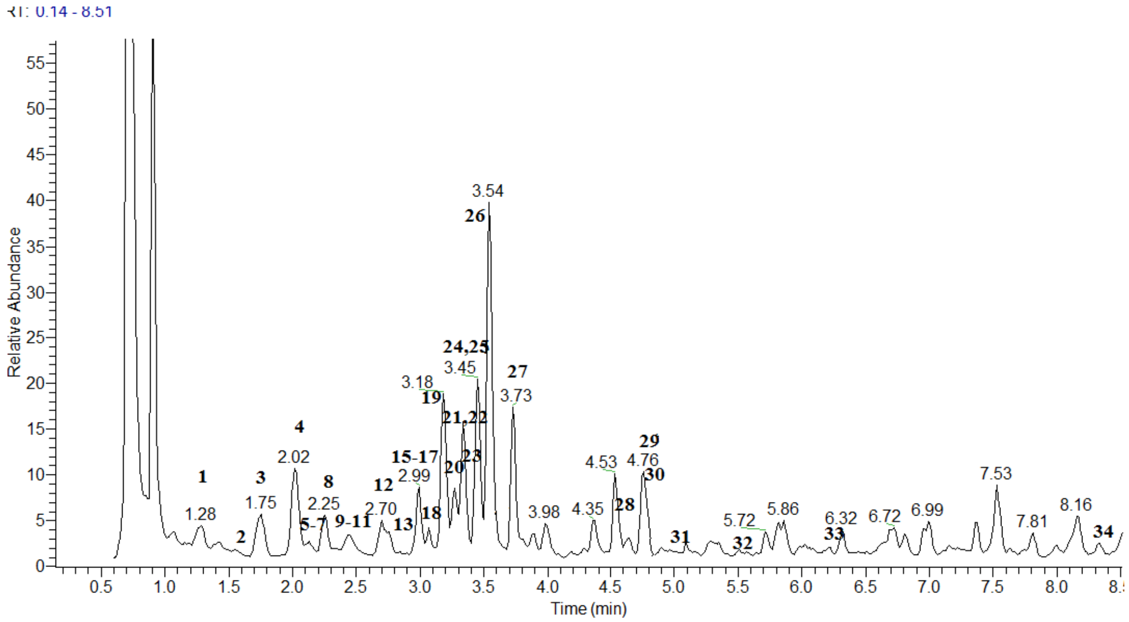

Figure S1. Extracted ion chromatograms of hydroxybenzoic and hydroxycinnamic acids and their derivatives (for numbers and fragmentation patterns, see Table S1).

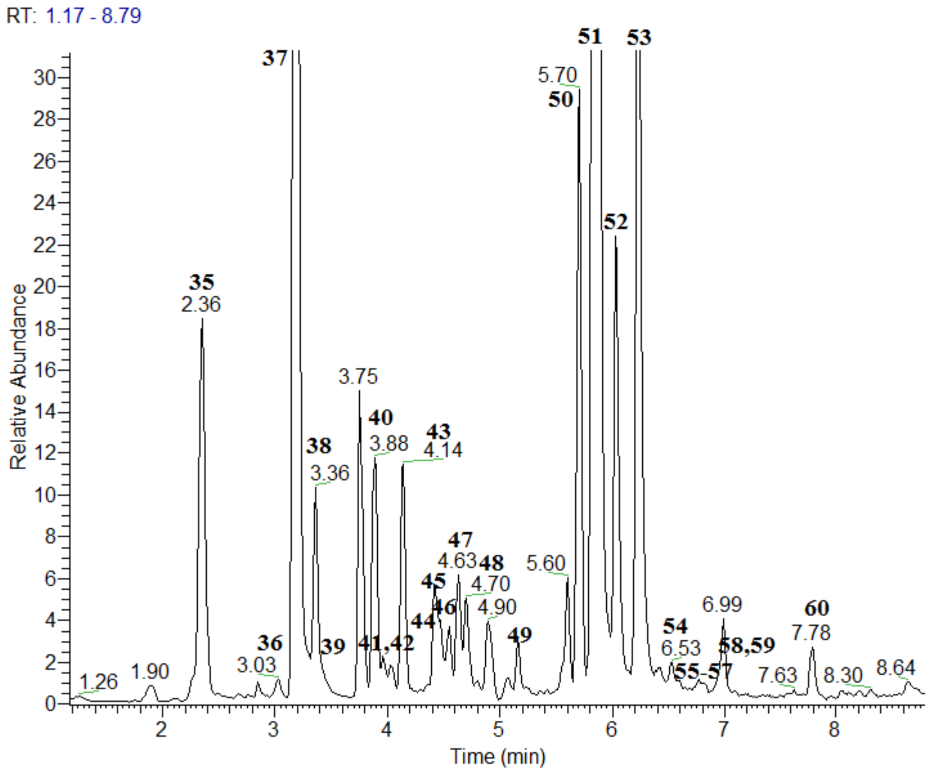

Figure S2. Extracted ion chromatograms of acylquinic acids (for numbers and fragmentation patterns, see Table S1).

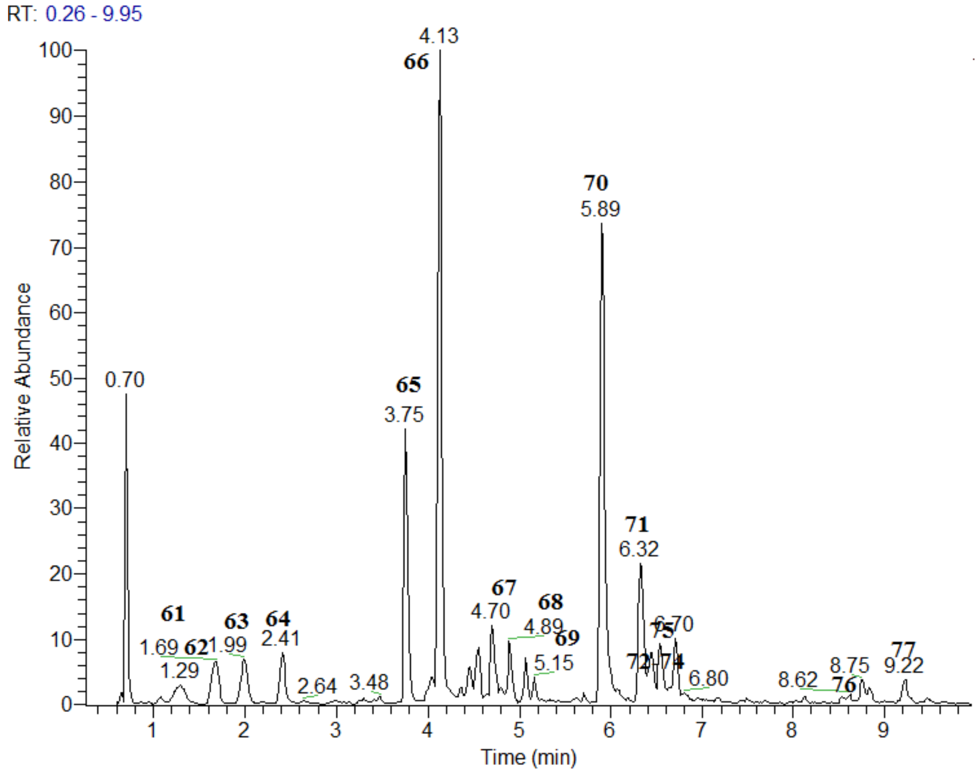

Figure S3. Extracted ion chromatograms of acylhexaric acids (for numbers and fragmentation patterns, see Table S1).

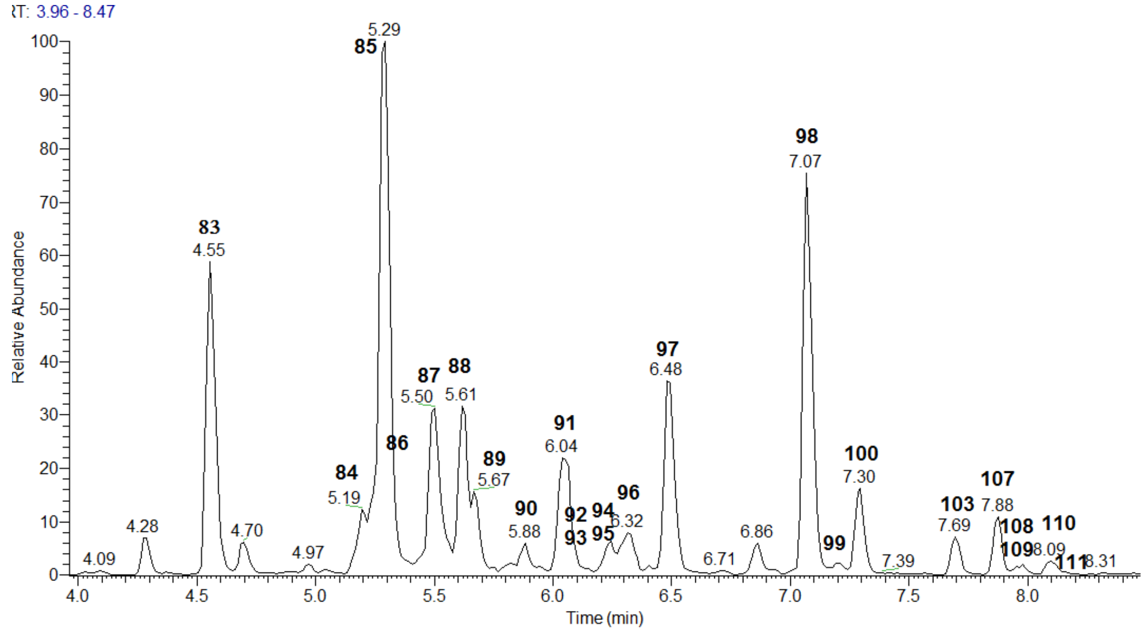

Figure S4. Extracted ion chromatograms of flavonoid glycosides (for numbers and fragmentation patterns, see Table S1).

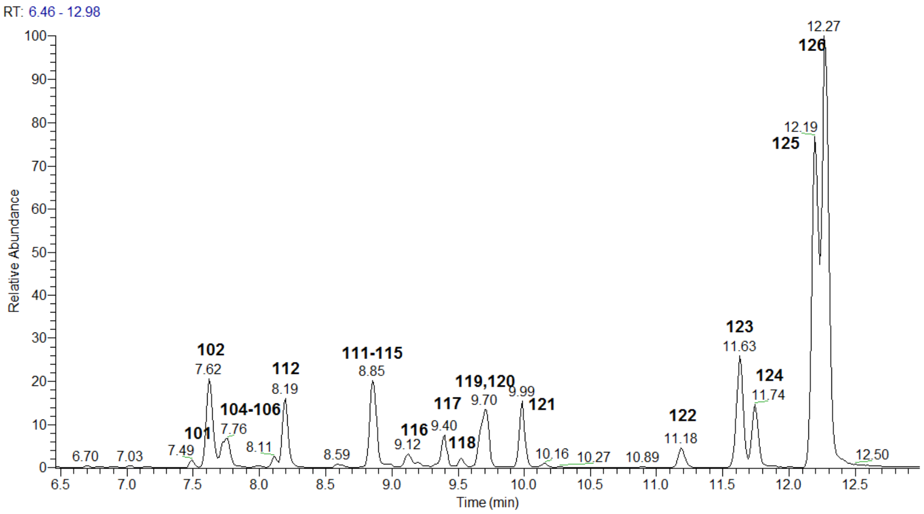

Figure S5. Extracted ion chromatograms of flavonoid aglycones (for numbers and fragmentation patterns, see Table S1).

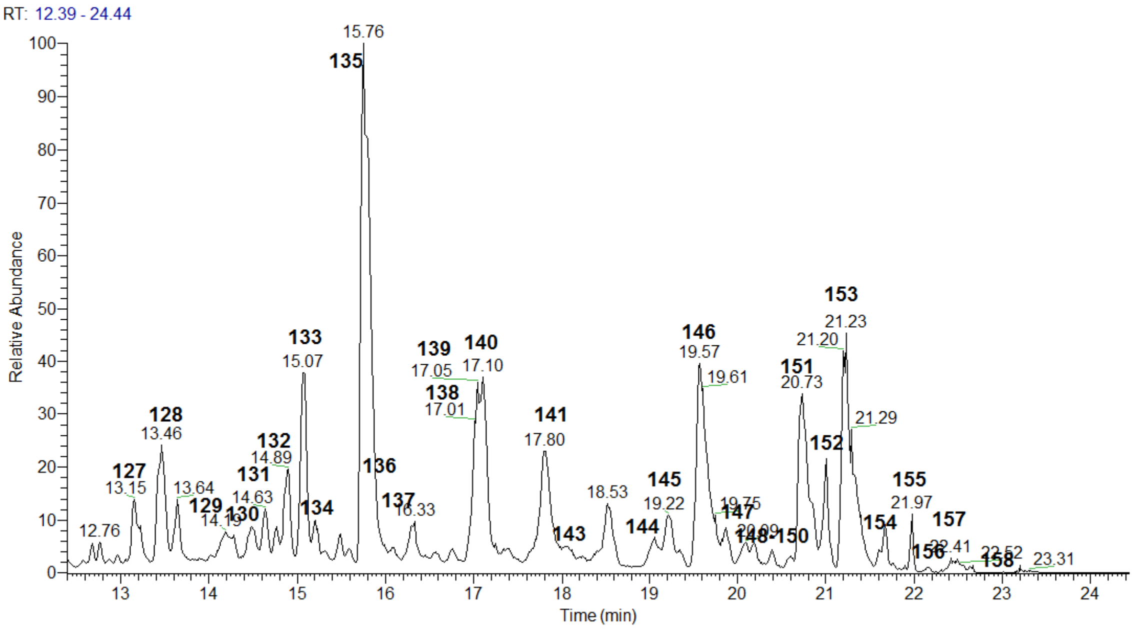

Figure S6. Extracted ion chromatograms of prenylated phloroglucinol α-pyrones (for numbers and fragmentation patterns, see Table S1).

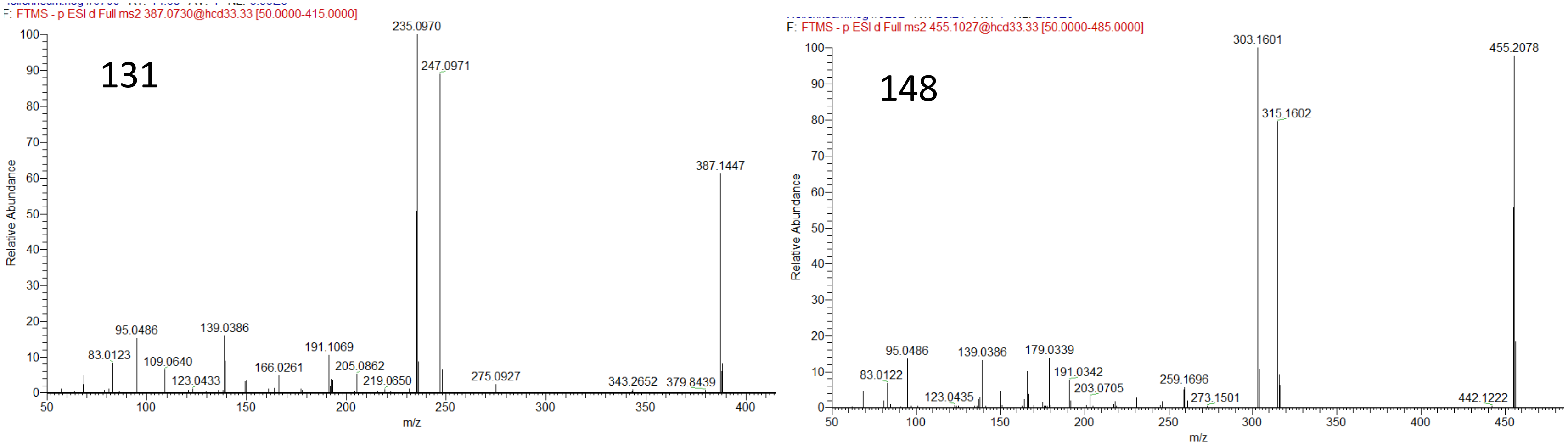

Figure S7. (-) ESI-MS/MS spectra of methylpyrones (MP) (For compound numbers and fragmentation patterns see Tab. S1).

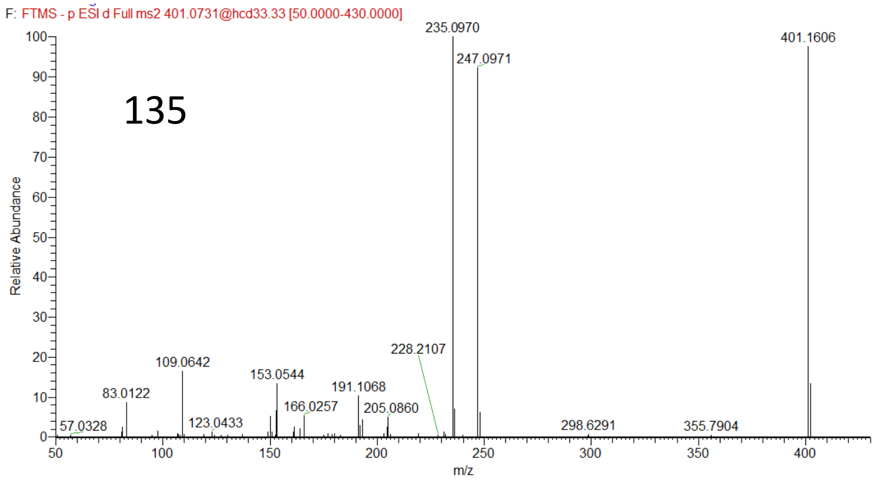
F: FTMS - p ESI d Full ms2 401.0731@hcd33.33 [50.0000-430.0000]
135
Relative Abundance
m/z
235.0970
247.0971
401.1606
228.2107
109.0642
153.0544
191.1068
83.0122
166.0257
205.0860
123.0433
57.0328
298.6291
355.7904

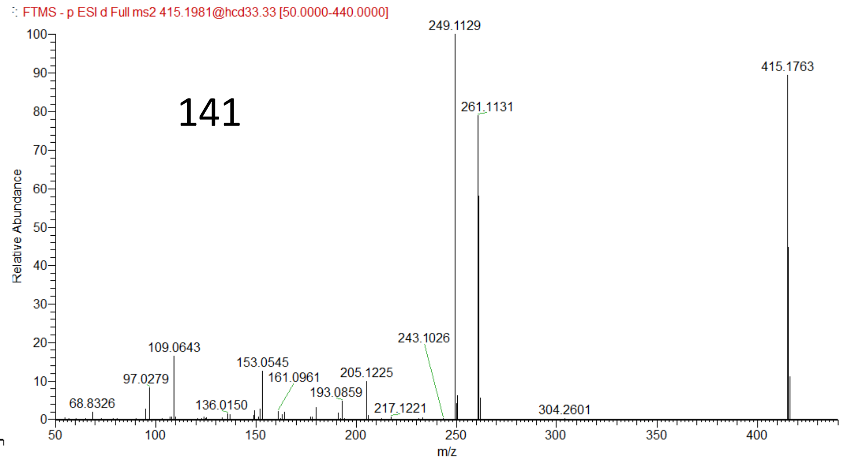
FTMS - p ESI d Full ms2 415.1981@hcd33.33 [50.0000-440.0000]
141
Relative Abundance
m/z
249.1129
415.1763
261.1131
243.1026
109.0643
153.0545
97.0279
161.0961
205.1225
193.0859
68.8326
136.0150
217.1221
304.2601

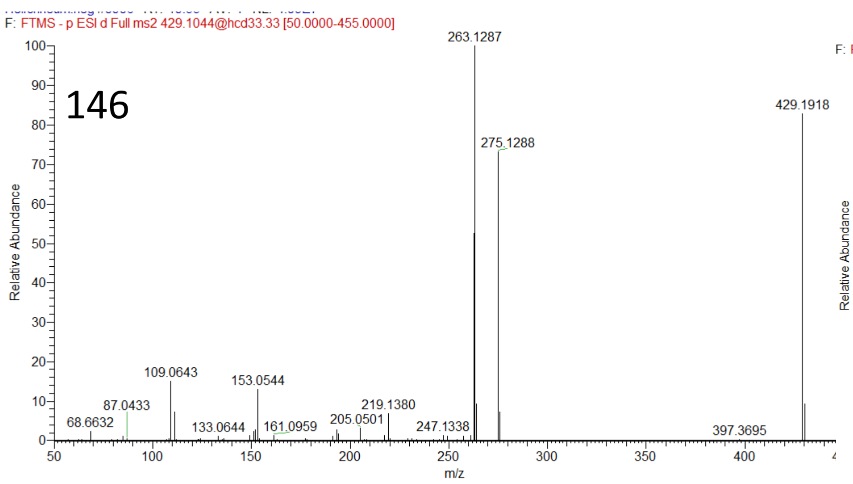
F: FTMS - p ESI d Full ms2 429.1044@hcd33.33 [50.0000-455.0000]
146
Relative Abundance
m/z
263.1287
429.1918
275.1288
109.0643
153.0544
87.0433
219.1380
68.6632
205.0501
133.0644
161.0959
247.1338
397.3695

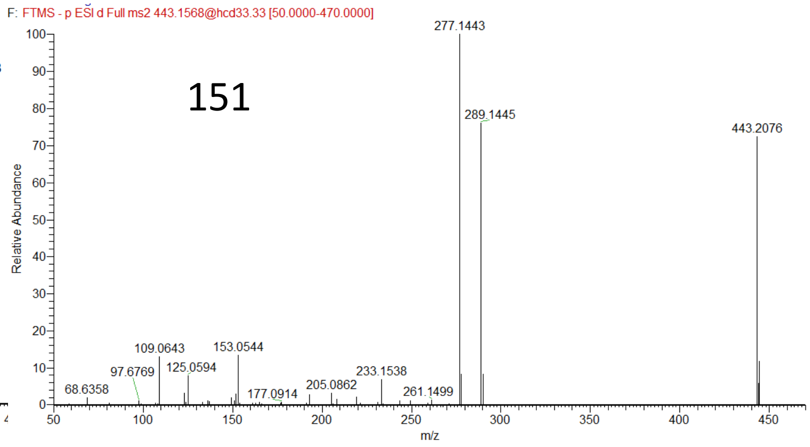
F: FTMS - p ESI d Full ms2 443.1568@hcd33.33 [50.0000-470.0000]
151
Relative Abundance
m/z
277.1443
289.1445
443.2076
109.0643
153.0544
97.6769
125.0594
233.1538
68.6358
205.0862
177.0914
261.1499

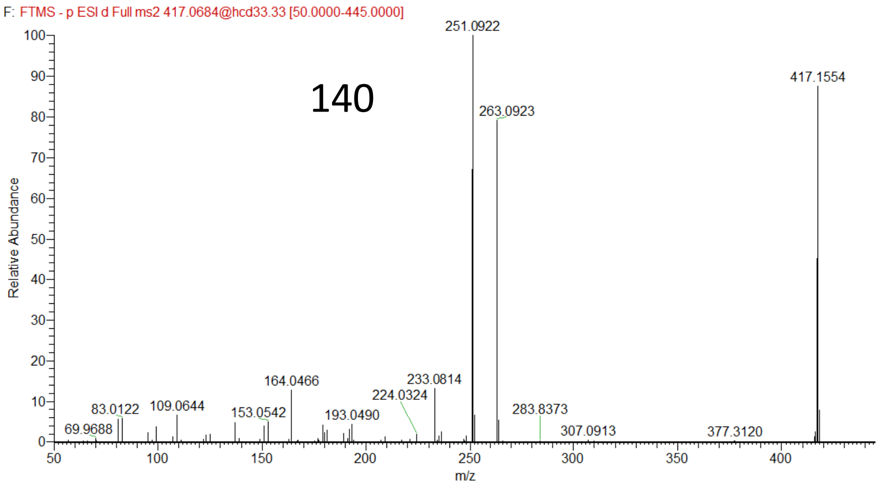
F: FTMS - p ESI d Full ms2 417.0684@hcd33.33 [50.0000-445.0000]
140
Relative Abundance
m/z
251.0922
263.0923
417.1554
164.0466
233.0814
224.0324
83.0122
109.0644
153.0542
193.0490
283.8373
69.9688
307.0913
377.3120

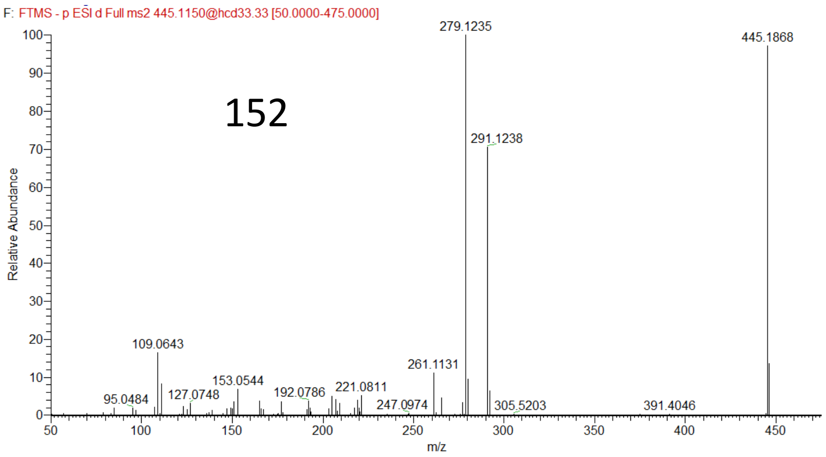
F: FTMS - p ESI d Full ms2 445.1150@hcd33.33 [50.0000-475.0000]
152
Relative Abundance
m/z
279.1235
445.1868
291.1238
109.0643
261.1131
153.0544
221.0811
95.0484
127.0748
192.0786
247.0974
305.5203
391.4046

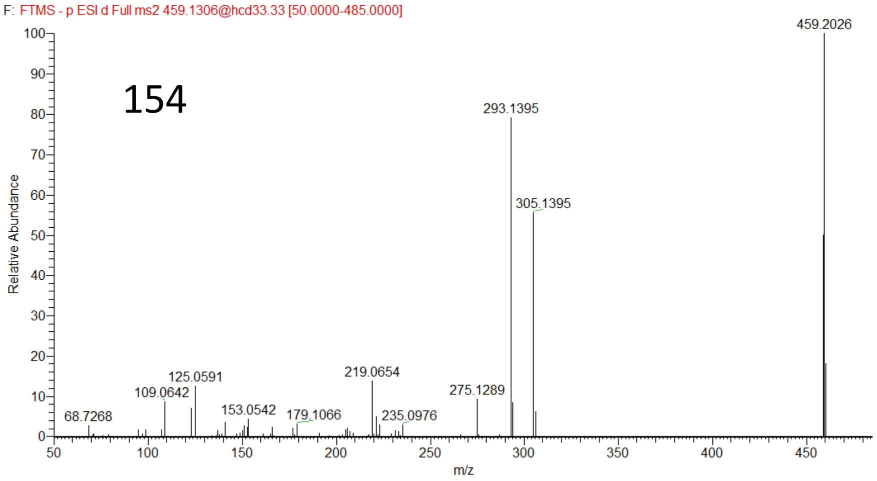
F: FTMS - p ESI d Full ms2 459.1306@hcd33.33 [50.0000-485.0000]
154
Relative Abundance
m/z
459.2026
293.1395
305.1395
125.0591
219.0654
109.0642
275.1289
153.0542
68.7268
179.1066
235.0976

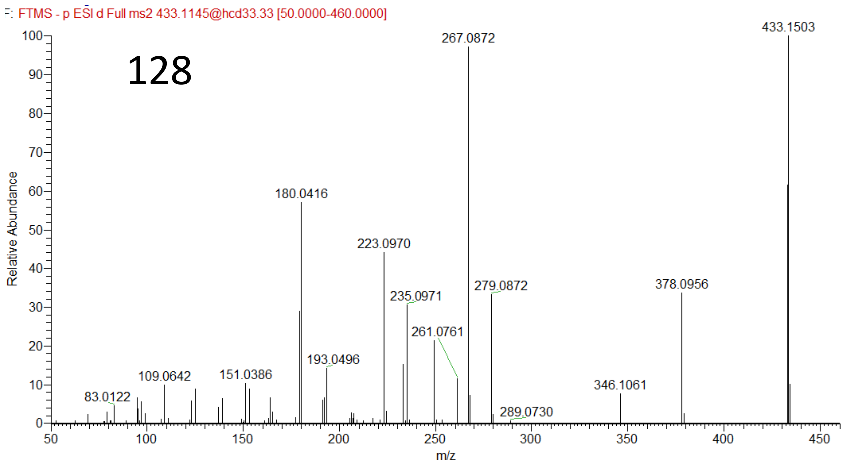

FTMS - p ESI d Full ms2 433.1145@hcd33.33 [50.0000-460.0000]
128
Relative Abundance
m/z
433.1503
267.0872
180.0416
223.0970
235.0971
279.0872
378.0956
261.0761
193.0496
151.0386
109.0642
83.0122
346.1061
289.0730

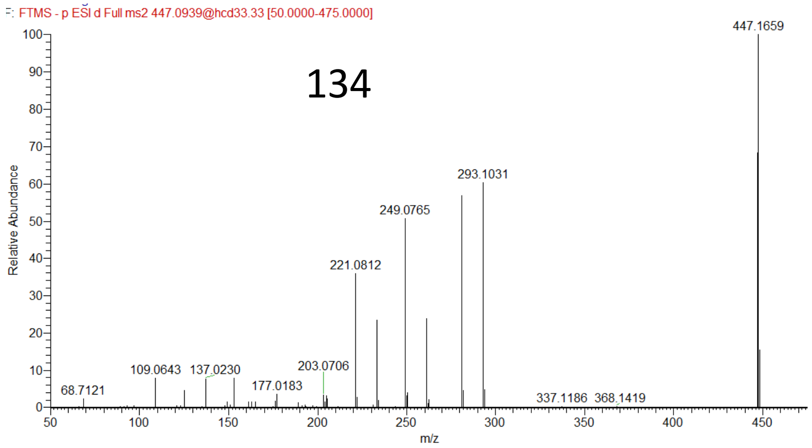

FTMS - p ESI d Full ms2 447.0939@hcd33.33 [50.0000-475.0000]
134
Relative Abundance
m/z
447.1659
293.1031
249.0765
221.0812
203.0706
109.0643
137.0230
177.0183
68.7121
337.1186
368.1419

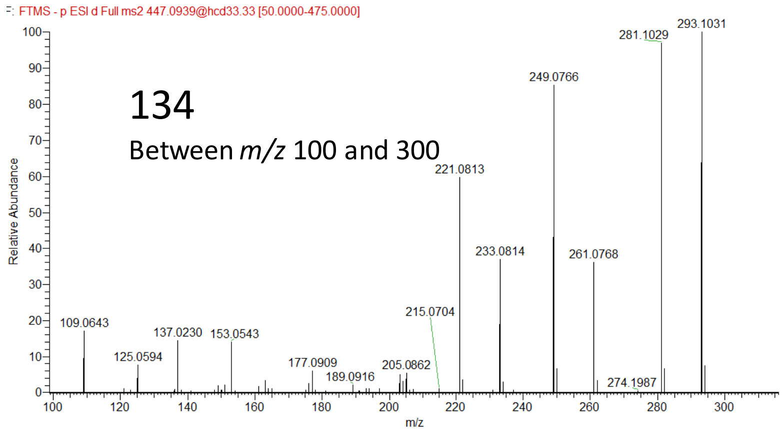

FTMS - p ESI d Full ms2 447.0939@hcd33.33 [50.0000-475.0000]
134
Between *m/z* 100 and 300
Relative Abundance
m/z
293.1031
281.1029
249.0766
221.0813
233.0814
261.0768
215.0704
109.0643
137.0230
153.0543
125.0594
177.0909
205.0862
189.0916
274.1987

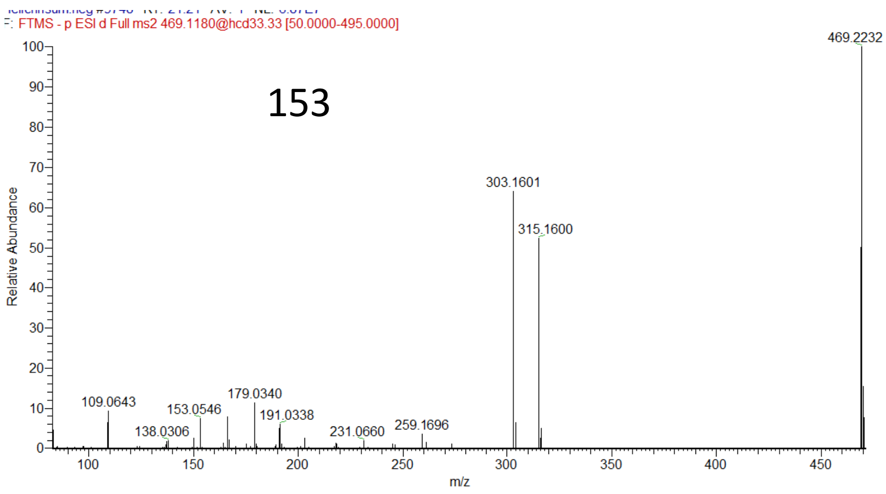

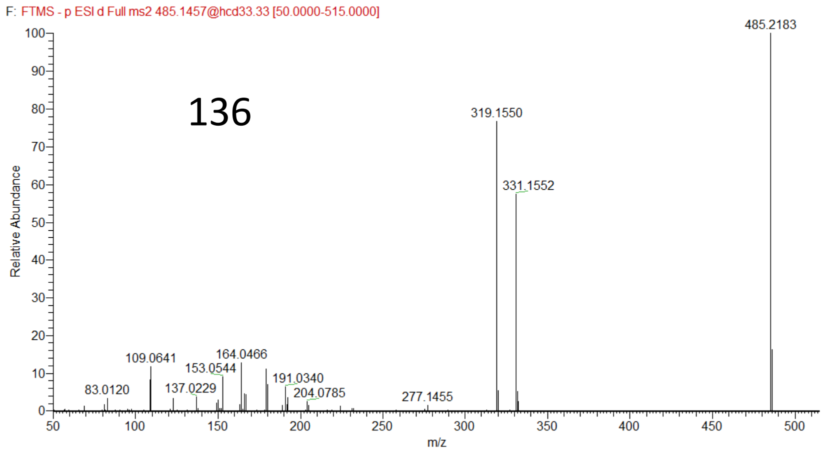

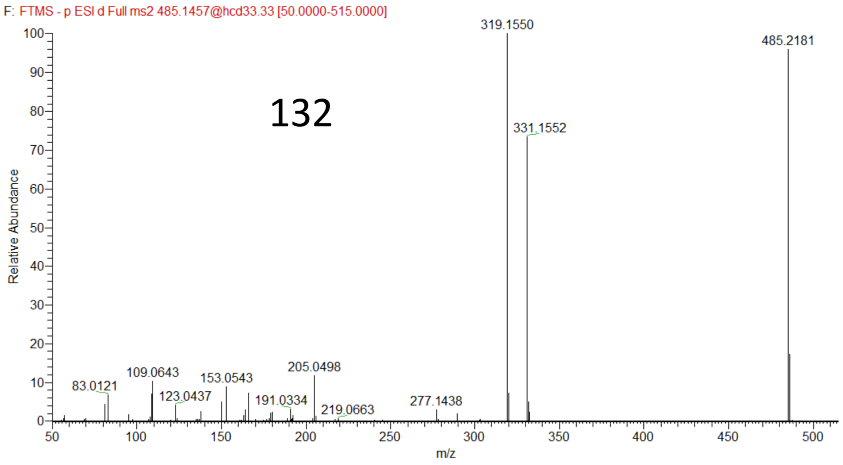

Figure S8. (-) ESI-MS/MS spectra of ethylpyrones (EP) (continue) (For compound numbers and fragmentation patterns see Tab. S1).

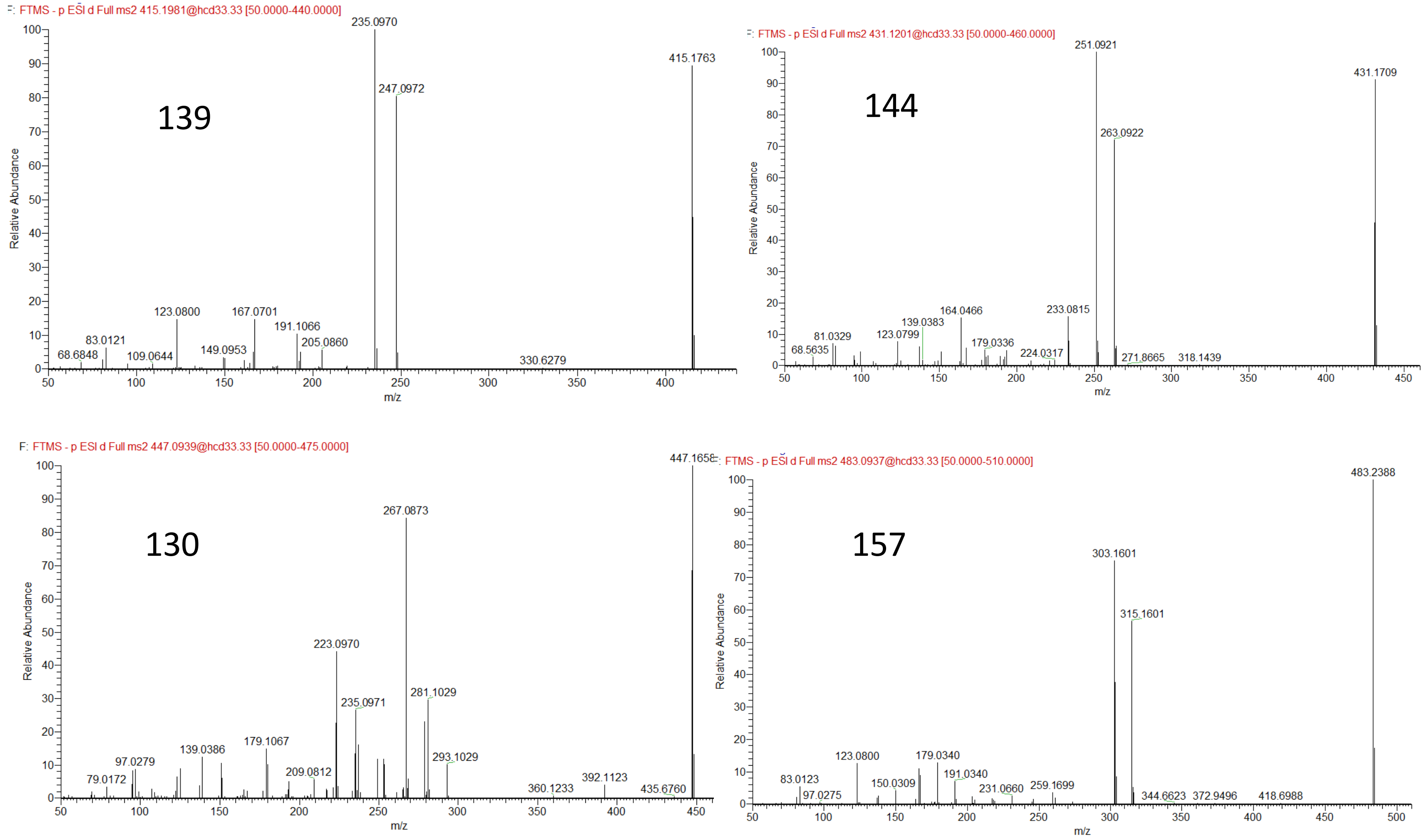

Figure S9. (-) ESI-MS/MS spectra of isopropylpyrones (IPP) (For compound numbers and fragmentation patterns see Tab. S1).

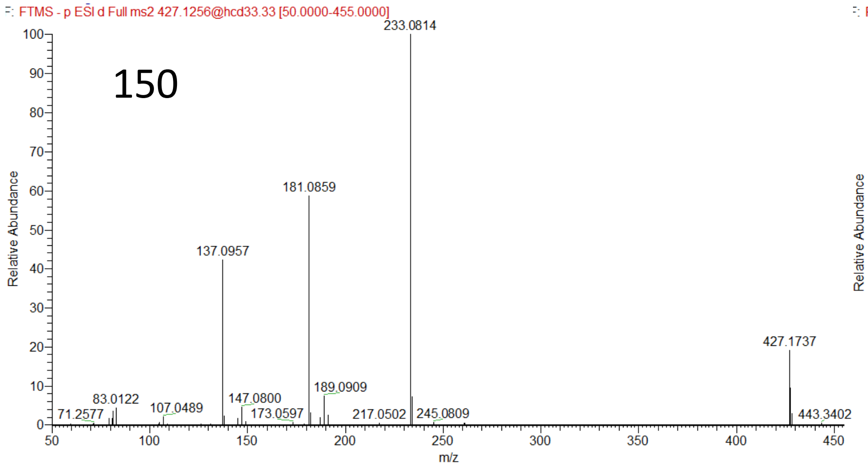

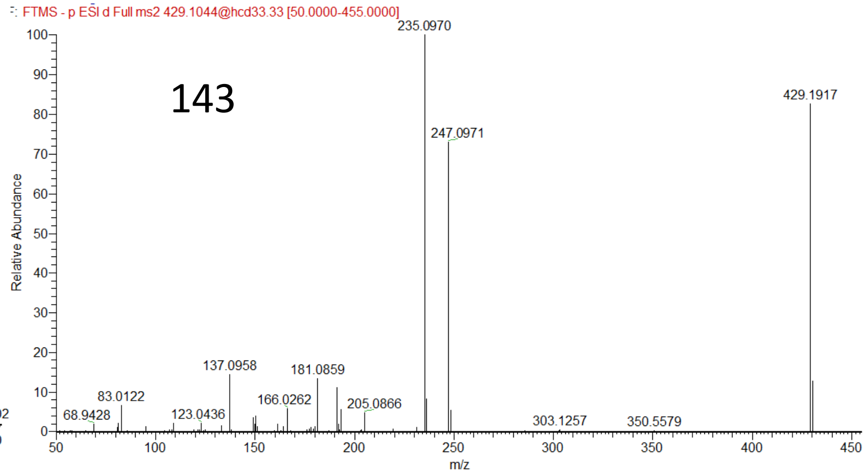

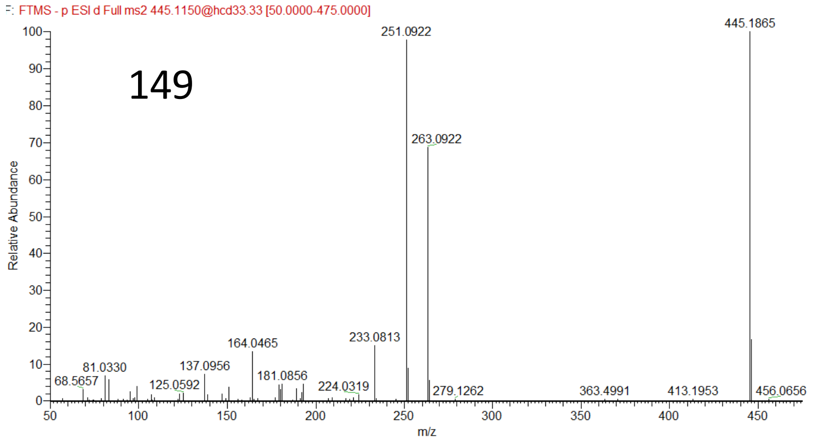

Figure S10. (-) ESI-MS/MS spectra of 1-methyl-propylpyrones (MPP) (For compound numbers and fragmentation patterns see Tab. S1).

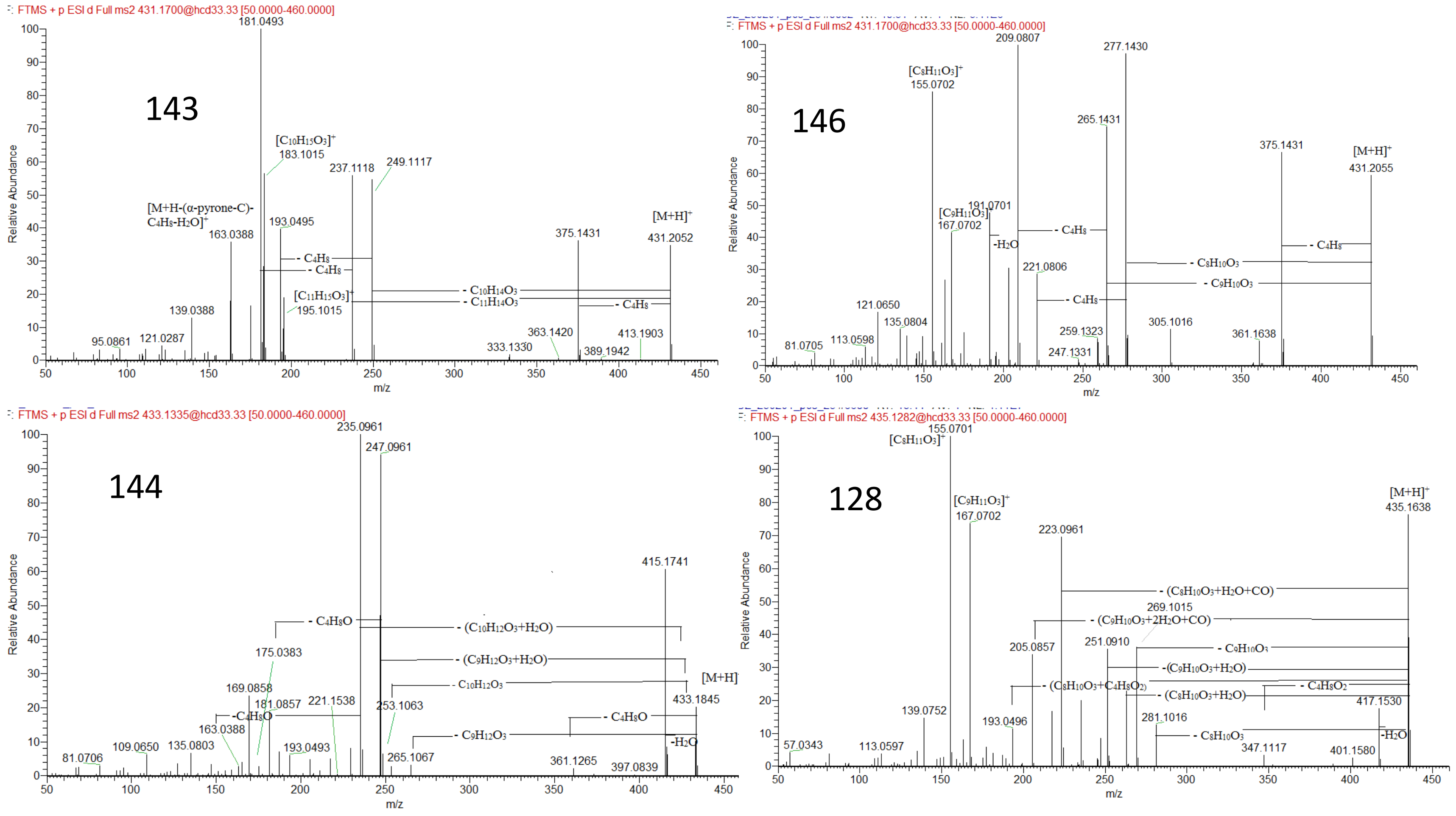

Figure S11. (+) ESI-MS/MS spectra of compounds **143, 144**, **146** and **128.**

Table S1. Secondary metabolites in *Helichrysum italicum* methanol-aqueous extract.

№	Identified/tentatively annotated compound	Molecular formula	Exact mass [M-H]⁻	Fragmentation pattern in (-) ESI-MS/MS	t_R (min)	Δ ppm	Level of confidence	References
	**Hydroxybenzoic, hydroxycinnamic and phenylethanoid glycosides**							
1.	hydroxybenzoic acid-*O*-hexoside	$C_{13}H_{16}O_8$	299.0778	299.0763 (0.2), 137.0229 (100), 93.0329 (65.8),	1.28	-3.279	2	[1]
2.	protocatechuic acid-*O*-hexoside	$C_{13}H_{16}O_9$	315.0727	315.0723 (100), 153.0181 (28.2), 152.0101 (60.2), 123.0073 (2.7), 109.0286 (11.7), 108.0201 (88.5)	1.68	0.115	2	[1]
3.	vanillic acid-*O*-hexoside	$C_{14}H_{18}O_9$	329.0875	329.0868 (1.8),167.0337 (100), 152.0102 (22.9), 123.0437 (13.8), 108.0201 (35.6)	1.75	-0.198	2	[1]
4.	protocatechuic acid[a]	$C_7H_6O_4$	153.0181	153.0179 (15.2), 109.0279 (100), 91.0173 (0.9), 81.0328 (1.2)	2.02	-1.392	1	[2]
5.	hydroxybenzoyl hexose[,b]	$C_{13}H_{16}O_8$	299.0778	299.0772 (91.8), 239.0556 (21.0), 209.0450 (6.2), 179.0330 (46.3), 137.0230 (100), 93.0330 (20.2)	2.04	0.002	2	
6.	protocatechuic acid-*O*-hexoside isomer	$C_{13}H_{16}O_9$	315.0727	315.0723 (48.1), 153.0543 (28.7), 123.0436 (54.7), 109.0279 (35.2)	2.11	1.729	2	
7.	*p*-hydroxyphenylacetic acid *O*-hexoside[b]	$C_{14}H_{18}O_8$	313.0932	313.0932 (100), 151.0387 (24.2), 123.0439 (7.7), 107.0488 (19.2)	2.12	0.988	2	
8.	syringic acid-*O*-hexoside[,b]	$C_{15}H_{20}O_{10}$	359.0985	359.0994 (8.4), 197.0446 (100), 182.0209 (19.9), 153.0544 (15.21), 138.0308 (26.4), 123.0072 (39.99)	2.24	0.036	2	
9.	caffeic acid-*O*-hexoside	$C_{15}H_{18}O_9$	341.0871	341.0871 (21.8), 281.0674 (4.5), 251.0553 (2.7), 221.0447 (3.3), 179.0339 (100), 161.0232 (39.0), 135.0437 (59.8)	2.41	-1.980	2	
10.	hydroxybenzoic acid-*O*-hexoside isomer	$C_{13}H_{16}O_8$	299.0778	299.0770 (13.0), 137.0229 (100), 93.0331 (0.1)	2.44	0.800	2	
11.	vanillyl *O*-hexose[b]	$C_{14}H_{18}O_9$	329.0875	329.0878 (100), 269.0669 (6.9), 239.0563 (3.4), 209.0448 (31.0), 167.0337 (53.2), 152.0099 (4.5), 123.0435 (8.0), 108.0200 (2.7)	2.47	0.075	2	
12.	esculetin-*O*-hexoside[b]	$C_{15}H_{15}O_9$	339.0724	339.0720 (2.7), 177.0181 (100), 149.0230 (1.1), 133.0279 (9.0), 105.0330 (3.8), 89.0381 (2.2),	2.70	0.515	2	
13.	syringyl-*O*-hexose[b]	$C_{15}H_{20}O_{10}$	359.0984	359.0983 (100), 269.0674 (1.7), 239.0556 (20.3), 197.0446 (36.7), 182.0209 (3.5), 153.0539 (1.8), 138.0307 (0.7), 123.0074 (2.3)	2.75	-0.278	2	
14.	4-hydroxybenzoic acid[a]	$C_7H_6O_3$	137.0230	137.0229 (100), 119.0124 (2.2), 108.0200 (9.1), 93.0330 (3.0), 65.0379 (1.2)	2.83	-10.928	2	[1, 2]
15.	gentisic acid[a]	$C_7H_6O_4$	153.0181	153.0179 (51.7), 125.0227 (1.2), 123.0072 (23.6), 109.0282 (35.2), 108.0200 (100), 81.0329 (5.7)	2.98	-8.051	1	

16.	*p*-hydroxyphenylacetic acid *O*-hexoside isomer[b]	$C_{14}H_{18}O_8$	313.0929	313.0929 (15.2), 151.0386 (100), 123.0071(0.5), 109.0279 (3.8)	2.99	0.030	2	
17.	hydroxybenzoic acid-*O*-hexoside	$C_{13}H_{16}O_8$	299.0778	299.0770 (13.0), 137.0229 (100), 108.0200 (0.8), 93.0331 (0.1)	2.99	-0.704	2	[1]
18.	caffeic acid *O*-hexoside	$C_{15}H_{18}O_9$	341.0871	341.0875 (8.4), 179.0337 (100), 135.0436 (68.9), 107.0486 (0.8)	3.07	-0.602	2	
19.	quinic acid	$C_7H_{12}O_6$	191.0549	191.0550 (100), 173.0443 (1. 9), 155.0336 (0.2), 127.0385 (4.0), 111.0435 (1.9), 93.0330 (6.0), 85.0279 (17.3)	3.18	-5.817	2	[2]
20.	caffeic acid-*O*-hexoside	$C_{15}H_{18}O_9$	341.0871	341.0875 (8.4), 179.0337 (100), 135.0436 (68.9), 107.0486 (0.8)	3.27	-0.386	2	[1]
21.	coumaric acid-*O*-hexoside	$C_{15}H_{18}O_8$	325.0930	325.0918 (1.3), 163.0387 (100), 1445.0281 (4.1), 119.0487 (87.7)	3.34	-3.355	2	[1, 2]
22.	*p*-coumaric acid[a]	$C_9H_8O_3$	163.0389	163.0387 (6.2), 145.0125 (0.3), 135.0436 (0.4), 119.0486 (100)	3.35	-8.203	1	[2]
23.	vanillic acid *O*-hexoside isomer	$C_{14}H_{18}O_9$	329.0875	329.0873 (2.5), 167.0337 (100), 151.0019 (1.5), 123.0436 (21.8), 107.0120 (0.8)	3.37	0.252	2	[1]
24.	esculetin	$C_9H_6O_4$	177.0193	177.0181 (100), 149.0230 (2.9), 133.0280 (19.9), 105.0329 (11.0), 89.0380 (8.4)	3.46	4.982	2	[1]
25.	*p*-hydroxyphenylacetic acid[a,b]	$C_8H_8O_3$	151.0401	151.0387 (100), 109.0279 (14.4), 124.0150 (4.1)	3.46	-9.252	2	
26.	caffeic acid[a]	$C_9H_8O_4$	179.0339	179.0339 (18.3), 135.0437 (100), 117.0332 (0.5), 107.0487 (1.2)	3.54	-6.044	1	[2]
27.	dehydrochorismic acid[b]	$C_{10}H_8O_6$	223.0248	223.0232 (1.3), 179.0338 (67.0), 153.0180 (23.5), 135.0437 (36.7), 109.0279 (100)	3.73	-7.180	2	
28.	*m*-coumaric acid[a,b]	$C_9H_8O_3$	163.0389	163.0388 (7.6), 119.0487 (100)	4.56	-7.958	1	
29.	syringic acid[a]	$C_9H_{10}O_5$	197.0446	197.0446 (45.5), 179.0339 (1.1), 167.0334 (0.4), 153.0543 (100)	4.76	-4.703	1	[2]
30.	vanillic acid[a,b]	$C_8H_8O_4$	167.0338	167.0337 (100), 152.0102 (24.6), 123.0436 (3.9), 95.0121 (3.3)	4.78	-7.615	1	
31.	*o*-coumaric acid[a,b]	$C_9H_8O_3$	163.0389	163.0388 (8.0), 135.0438 (0.6), 119.0487 (100)	5.01	-7.958	1	
32.	scopoletin	$C_{10}H_8O_4$	191.0350	191.0340 (22.4), 176.0103 (100), 148.0151 (15.4), 120.0200 (1.2), 104.0251 (19.0)	5.06	-5.088	2	[1]
33.	caffeic acid *O*-(hydroxyisovaleryl)-hexoside[b]	$C_{20}H_{26}O_{11}$	441.1402	441.1402 (23.2), 323.0782 (0.8), 179.0338 (100), 135.0437 (68.1), 107.0487 (0.6),	5.58	0.125	2	
34.	salicylic acid[a]	$C_7H_6O_3$	137.0230	137.0230 (33.1), 108.0201 (3.3), 93.0330 (100)	6.27	-10.344	1	[2]

35.	caffeic acid *O*-(hydroxybensoyl)-hexoside[b]	$C_{22}H_{22}O_{11}$	461.1089	461.1090 (21.7), 323.0780 (4.9), 179.0339 (97.0), 137.0229 (100), 135.0437 (85.5), 93.0330 (38.0)	8.26	0.142	1	
				**Mono-, diacyl- and triacylquinic acids**				
36.	neochlorogenic (3-caffeoylquinic) acid[a,b]	$C_{16}H_{18}O_9$	353.0867	353.0880 (40.4), 191.0551 (100), 179.0339 (65.2), 173.0442 (3.3), 161.0231 (3.8), 135.0437 (45.3), 111.0433 (2.0), 93.0330 (4.1), 85.0279 (7.5)	2.35	0.495	1	
37.	3-*p*-coumaroylquinic acid	$C_{16}H_{18}O_8$	337.0928	337.0933(14.3), 191.0551 (28.7), 173.0441 (5.5), 163.0387 (100), 135.0435 (1.3), 119.0486 (30.3), 111.0432(1.5), 93.0329 (2.6), 85.0278 (3.0)	3.02	1.096	2	
38.	chlorogenic (5-caffeoylquinic) acid[a]	$C_{16}H_{18}O_9$	353.0874	353.0880 (4.8), 191.0550 (100), 179.0335 (1.0), 161.0231 (1.6), 127.0384 (1.8), 111.0436 (9.7), 93.0329 (2.6), 85.0278 (6.5)	3.18	0.665	1	[1, 2]
39.	4-caffeoylquinic acid	$C_{16}H_{18}O_9$	353.0878	353.0880 (32.0), 191.0551 (60.6), 179.0339 (70.9), 173.0445 (100), 155.0337 (4.8), 135.0437 (51.7), 111.0436 (2.8), 93.0330 (20.6), 85.0278 (9.0)	3.35	-0.100	2	[1]
40.	3-feruloylquinic acid	$C_{17}H_{20}O_9$	367.1034	367.1035 (20.8), 193.0496 (100), 149.0594 (2.8), 134.0358 (53.2), 127.0388 (0.6), 111.0438 (1.0), 93.0329 (1.7)	3.43	..	2	[2]
41.	5-caffeoylquinic acid isomer	$C_{16}H_{18}O_9$	353.0874	353.0880(6.4), 191.0550 (100), 179.0341 (0.9), 173.0446 (0.9), 161.0233 (2.1), 127.0383 (1.7), 93.0330 (2.6), 85.0279 (8.2)	3.88	0.580	2	[1]
42.	5-*p*-coumaroylquinic acid	$C_{16}H_{18}O_8$	337.0928	337.0932 (7.8), 191.0550 (100), 173.0442 (6.6), 163.0389 (5.9), 145.0281 (1.5), 119.0487 (5.2), 93.0328 (18.5), 85.0277 (4.4)	3.96	0.829	2	[1]
43.	3-caffeoyl-5-hydroxy-dihydrocaffeoylquinic acid[b]	$C_{25}H_{26}O_{13}$	533.1288	533.1302 (68.2), 371.0992 (2.7), 353.0874 (17.8), 335.0778 (4.0), 191.0551 (100), 179.0338 (42.9), 173.0444 (14.4), 161.0233 (7.3), 135.0437 (54.8), 111.0435 (2.0), 93.0329 (8.0), 85.0279 (9.3)	4.03	0.236	2	
44.	1, 3- dicaffeoylquinic acid[b]	$C_{25}H_{24}O_{12}$	515.1190	515.1196 (82.0), 353.0879 (38.9), 335.0772 (12.2), 191.0550 (100), 179.0338 (69.5), 161.0230 (7.7), 135.0436 (61.4), 111.0438 (3.5), 93.0328 (5.3)85.0278 (7.2)	4.14	0.137	2	
45.	3-caffeoyl-4- hydroxy-dihydrocaffeoylquinic acid[b]	$C_{25}H_{26}O_{13}$	533.1288	533.1312 (100), 371.0615 (13.7), 353.0875 (6.7), 335.0773 (11.2), 191.0551 (10.4), 179.0339 (21.9), 173.0444 (92.0), 161.0231 (16.1), 135.0437 (71.5), 127.0385 (2.0), 111.0437 (1.5), 93.0330 (19.0)	4.37	2.1	2	
46.	5-feruloylquinic acid	$C_{17}H_{20}O_9$	367.1034	367.1035 (16.7), 193.0497 (5.7), 191.0551 (100), 173.0444 (12.0), 155.0338 (0.4), 134.0359 (10.1), 111.0437 (3.5), 93.0329 (25.2), 85.0278 (5.1)	4.42	-0.096	2	[1]

47.	1-caffeoyl-3-hydroxy-dihydrocaffeoylquinic acid[b]	$C_{25}H_{26}O_{13}$	533.1288	533.1368 (17.8), 371.0991 (26.6), 353.0878 (5.6), 335.0771 (2.9), 191.0551 (26.1), 179.0338 (10.2), 173.0445 (12.5), 161.0235 (3.4), 135.0436 (100), 93.0330 (3.9), 85.0278 (23.7)	4.45	12.710	2	
48.	5-*p*-coumaroylquinic acid isomer	$C_{16}H_{18}O_8$	337.0928	337.0929 (7.4), 191.0550 (100), 173.0444 (1.9), 163.0388 (1.8), 127.0384 (1.5), 111.0435 (1.0), 93.0329 (4.7), 85.0277 (6.7)	4.63	0.028	2	[1]
49.	4-feruloylquinic acid[b]	$C_{17}H_{20}O_9$	367.1034	367.1034 (95.0), 193.0496 (10.9), 191.0562 (1.7), 173.0444 (72.13), 163.0389 (2.2), 134.0359 (22.2), 111.0436 (14.9), 93.0329 (100)	4.69	-0.260	2	
50.	1, 3-dicaffeoylquinic acid-hexoside	$C_{31}H_{34}O_{17}$	677.1723	677.1730 (81.2), 515.1407 (82.0), 353.0876 (17.2), 341.0877 (30.0), 323.0772 (19.9), 191.0551 (82.1), 179.0339 (98.9), 173.0441 (3.8), 161.0230 (29.6), 135.0437 (100), 93.0331 (7.1), 85.0279 (6.9)	5.15	0.956	2	[1]
51.	3,4-dicaffeoylquinic acid[a]	$C_{25}H_{24}O_{12}$	515.1190	515.1196 (100), 353.0877 (12.3), 335.0780 (5.0), 203.0334 (0.4), 191.0551 (24.9), 179.0339 (41.6), 173.0444 (46.7), 161.0232 (13.1), 135.0437 (44.2), 127.0386 (1.9), 111.0436 (3.4), 93.0329 (14.6), 85.0278 (3.1)	5.71	0.254	1	[1, 2]
52.	3,5-dicaffeoylquinic acid[a]	$C_{25}H_{24}O_{12}$	515.1189	515.1197 (19.6), 353.0879 (100), 335.0774 (1.5), 191.0551 (87.0), 179.0339 (38.8), 173.0445 (3.8), 161.0233 (4.7), 135.0437 (1.4), 111.0434 (1.5), 93.0330 (4.1), 85.0279 (7.5)	5.85	0.370	1	[1]
53.	1,3-dicaffeoylquinic acid malonyl[b]	$C_{28}H_{26}O_{15}$	601.1199	601.1207 (37.5), 557.1307 (16.1), 515.1199 (11.1), 439.0883 (26.8), 395.0983 (43.2), 233.0661 (100), 191.0551 (17.5), 179.0338 (14.1), 173.0443 (14.1), 161.0231 (11.1), 155.0334 (3.5), 135.0436 (15.6), 133.0280 (5.3), 127.0385 (1.4), 111.0435 (2.5), 93.0330 (6.3), 85.0278 (1.2), 59.0122 (91.9)	5.95	1.292	2	
54.	1,5-dicaffeoylquinic acid[a]	$C_{25}H_{24}O_{12}$	515.1190	515.1197 (28.5), 353.0878 (83.6), 335.0764 (2.5), 191.0551 (100), 179.0339 (47.4), 173.0445 (7.1), 161.0231 (7.3), 155.0338 (1.4), 135.0437 (56.5), 127.0385 (3.4), 111.0434 (1.9), 93.0330 (5.7), 85.0279 (9.2)	6.03	-0.096	1	[1]
55.	3, 5-dicaffeoylquinic acid malonyl[b]	$C_{28}H_{26}O_{15}$	601.1199	601.1199 (20.6), 557.1304 (13.8), 515.1189 (11.8), 439.0882 (9.9), 395.0982 (80.0), 353.0869 (0.5), 335.0772 (2.5), 233.0661 (100), 191.0551 (6.7), 179.0339 (5.4), 173.0443 (17.3), 161.0233 (8.5), 135.0436 (11.2), 133.0281 (3.4), 111.0435 (2.5), 93.0329 (5.6), 59.0122 (88.4)	6.15	-0.039	2	

56.	4,5-dicaffeoylquinic acid	$C_{25}H_{24}O_{12}$	515.1190	515.1196 (100), 353.0878 (64.2), 335.0765 (0.6), 229.0565 (0.6), 203.0345 (3.0), 191.0551 (34.0), 179.0338 (70.0), 173.0443 (85.5), 155.0336 (4.7), 135.0436 (51.0), 127.0383 (0.7), 111.0436 (3.0), 93.0329 (18.7), 85.0280 (3.2)	6.24	0.254	2	[1]
57.	3, 4-dicaffeoylquinic acid malonyl[b]	$C_{28}H_{26}O_{15}$	601.1199	601.1202 (20.6), 557.1302 (11.0), 515.1196 (4.5), 439.0883 (15.8), 395.0984 (46.1), 353.0877 (0.8), 233.0661 (100), 191.0551 (11.6), 179.0339 (7.4), 173.0443 (81.1), 161.0231 (6.4), 155.0336 (4.7), 135.0438 (13.7), 133.0280 (4.3), 111.0438 (4.6), 93.0328 (17.6), 59.0122 (27.4)	6.32	0.577	2	
58.	4, 5-dicaffeoylquinic acid malonyl[b]	$C_{28}H_{26}O_{15}$	601.1199	601.1203 (8.4), 557.1302 (10.0), 515.1190 (10.9), 439.0883 (25.5), 395.0981 (100), 353.0878 (16.1), 335.0777 (16.1), 233.0661 (81.7), 191.0551 (44.8), 179.0338 (25.9), 173.0443 (74.7), 161.0232 (11.2), 155.0337 (5.1), 135.0437 (39.1), 111.0435 (4.2), 93.0329 (16.9), 85.0279 (3.9), 59.0122 (29.0)	6.51	0.677	2	
59.	3-*p*-coumaroyl-5-caffeoylquinic acid[b]	$C_{25}H_{24}O_{11}$	499.1251	499.1259 (28.4), 353.0869 (1.8), 337.0929 (81.4), 335.0773 (2.0), 191.0552 (21.9), 179.0341 (1.2), 173.0443 (8.6), 163.0387 (100), 135.0436 (3.5), 119.0487 (35.6), 93.0330 (4.4), 85.0276 (1.5)	6.53	2.535	2	
60.	3-feruloyl-5-caffeoylquinic acid	$C_{26}H_{26}O_{12}$	529.1356	529.1347 (12.6), 367.1034 (100), 353.0889 (1.4), 335.0789 (0.7), 193.0496 (92.8), 191.0551 (28.3), 179.0342 (2.2), 173.0443 (11.8), 161.0233 (3.9), 155.0336 (2.4), 134.0358 (62.5), 111.0433 (2.2), 93.0329 (0.7), 85.0278 (1.8)	6.83	-0.849	2	[1]
61.	3-caffeoyl-5-feruloylquinic acid[b]	$C_{26}H_{26}O_{12}$	529.1356	529.1356 (47.0), 367.1034 (59.1), 353.0879 (44.1), 335.0770 (1.8), 193.0497 (42.5), 191.0551 (100), 179.0339 (34.3), 173.0444 (16.9), 161.0232 (8.0), 155.0334 (2.4), 135.0438 (36.1), 134.0358 (36.1), 111.0437 (3.4), 93.0329 (16.5), 85.0278 (7.2)	6.90	0.776	2	
62.	4-*p*-coumaroyl-5-caffeoylquinic acid[b]	$C_{25}H_{24}O_{11}$	499.1252	499.1225 (26.5), 353.0879 (1.2), 337.0930 (63.0), 191.0549 (6.5), 173.0443 (100), 163.0388 (19.1), 135.0436 (4.4), 133.0278 (1.4), 127.0385 (1.4), 121.0279 (45.9), 119.0486 (11.2), 111.0435 (3.3), 93.0329 (24.5), 85.0279 (1.9)	6.94	-4.117	2	
63.	4-feruloyl-5-caffeoylquinic acid	$C_{26}H_{26}O_{12}$	529.1356	529.1351 (18.5), 367.1034 (67.2), 353.0881 (0.6), 335.0780 (0.4), 193.0497 (17.8), 173.0443 (100),	7.09	-0.037	2	[1]

				155.0337 (4.2), 137.0231 (4.4), 134.0359 (16.3), 127.0386 (0.7), 111.0437 (2.6), 93.0330 (23.8)				
64.	4-caffeoyl-5-feruloylquinic acid[b]	$C_{26}H_{26}O_{12}$	529.1356	529.1354 (7.2), 367.1032 (21.7), 353.0879 (55.4), 335.0784 (1.9), 203.0345 (1.8), 191.0551 (72.8), 179.0338 (66.8), 173.0443 (100), 161.0231 (11.0), 135.0437 (60.9), 111.0435 (5.0), 93.0329 (34.5), 85.0278 (7.6), 134.0359 (8.8)	7.18	0.474	2	
65.	3,4,5-tricaffeoylquinic acid[a]	$C_{34}H_{30}O_{15}$	677.1528	677.1523 (100), 515.1199 (50.2), 353.0880 (44.9), 335.0772 (14.7), 299.0559 (0.6), 255.0667 (0.8), 203.0342 (0.9), 191.0552 (41.4), 179.0339 (71.4), 173.0444 (87.5), 161.0232 (22.2), 135.0437 (74.3), 111.0437 (5.6), 93.0330 (20.3), 85.0279 (3.8)	7.78	1.605	1	
				**Caffeoylhexaric acids**				
66.	caffeoylhexaric acid[b] 1	$C_{15}H_{16}O_{11}$	371.0620	371.0625 (36.1), 209.0295 (100), 179.0338 (3.2), 173.0086 (0.7), 161.0228 (2.2), 147.0286 (4.3), 135.0437 (0.8), 129.0178 (4.0), 111.0076 (2.4), 93.0329 (9.7), 85.0278 (48.7)	1.29	1.470	2	
67.	caffeoylhexaric acid[b] 2	$C_{15}H_{16}O_{11}$	371.0620	371.0612 (3.1), 209.0295 (100), 191.0188 (21.5), 179.0337 (2.2), 173.0079 (1.6), 147.0286 (4.9), 135.0437 (3.9), 129.0182 (3.1), 111.0074 (2.3), 85.0278 (43.8)	1.69	-1.979	2	
68.	caffeoylhexaric acid[b] 3	$C_{15}H_{16}O_{11}$	371.0620	371.0605 (3.0), 209.0294 (100), 173.0079 (1.6), 147.0286 (3.7), 135.0436 (2.0), 129.0178 (3.9), 111.0066 (2.0), 85.0274 (42.0)	1.99	-4.028	2	
69.	caffeoylhexaric acid[b] 4	$C_{15}H_{16}O_{11}$	371.0620	371.0594 (2.5), 209.0294 (100), 191.0187 (21.0), 147.0282 (3.2), 135.0437 (1.5), 129.0176 (2.1), 111.0071 (2.2), 85.0278 (36.2)	2.41	-6.956	2	
70.	dicaffeoylhexaric acid 1	$C_{24}H_{22}O_{14}$	533.0937	533.0938 (13.0), 371.0620 (95.9), 353.0513 (2.3), 209.0295 (100), 191.0188 (26.2), 179.0333 (3.3), 161.0236 (0.6), 173.0081 (0.7), 147.0289 (4.2), 135.0439 (5.2), 129.0177 (5.3), 111.0072 (2.9), 85.0279 (51.5)	3.75	0.134	2	[1]
71.	dicaffeoylhexaric acid[b] 2	$C_{24}H_{22}O_{14}$	533.0937	533.0893 (13.4), 371.0618 (85.3), 209.0294 (100), 191.0186 (23.6), 179.0341 (6.3), 173.0081 (0.8), 147.0285 (3.0), 135.0436 (11.3), 129.0178 (6.6), 111.0072 (4.1), 85.0278 (48.4)	4.13	-8.213	2	
72.	dicaffeoylhexaric acid[b] 3	$C_{24}H_{22}O_{14}$	533.0937	533.0862 (15.4), 371.0610 (89.8), 353.0517 (4.5), 335.0406 (0.5), 209.0295 (100), 191.0188 (28.8), 179.0340 (4.9), 161.0232 (0.9), 147.0283 (4.0), 135.0437 (14.6), 129.0179 (6.9), 111.0074 (2.8), 93.329 (4.2), 85.0278 (48.2)	4.71	-14.066	2	

73.	dicaffeoylhexaric acid[b] 4	$C_{24}H_{22}O_{14}$	533.0937	533.0859 (15.4), 371.0615 (76.1), 353.0512 (3.3), 209.0294 (100), 191.0188 (25.7), 179.0341 (3.8), 173.0446 (4.6), 161.0234 (1.2), 147.0285 (3.2), 135.0437 (12.3), 129.0178 (6.6), 129.0178 (4.5), 111.0072 (2.7), 93.0329 (1.3), 85.0278 (49.3)	4.89	-14.516	2	
74.	dicaffeoylhexaric acid[b] 5	$C_{24}H_{22}O_{14}$	533.0937	533.0892 (16.1), 371.0619 (91.0), 353.0510 (0.3), 209.0295 (100), 191.0188 (31.9), 179.0339 (3.7), 161.0229 (2.7), 147.0285 (5.0), 135.0438 (8.7), 129.0178 (5.7), 111.0070 (3.1), 85.0278 (55.0)	5.15	-8.457	2	
75.	tricaffeoylhexaric acid 1	$C_{33}H_{28}O_{17}$	695.1254	695.1259 (24.2), 533.0938 (31.6), 371.0619 (19.4), 353.0510 (1.7), 209.0294 (100), 191.0148 (14.8), 179.0339 (3.8), 161.0233 (0.8), 147.0285 (3.5), 135.0437 (6.3), 129.0178 (6.1), 111.0072 (2.6), 85.0278 (37.9)	5.89	0.788	2	[1]
76.	tricaffeoylhexaric acid[b] 2	$C_{33}H_{28}O_{17}$	695.1254	695.1262 (24.3), 533.0942 (34.6), 371.0622 (20.3), 353.0510 (1.7), 209.0295 (100), 191.0148 (15.6), 179.0341 (2.9), 161.0230 (2.1), 147.0287 (4.7), 135.0436 (4.7), 129.0179 (5.3), 111.0072 (2.2), 85.0279 (43.7)	6.32	1.219	2	
77.	tricaffeoylhexaric acid[b] 3	$C_{33}H_{28}O_{17}$	695.1254	695.1262 (21.2), 533.0941 (44.3), 371.0620 (20.5), 209.0294 (100), 191.0188 (18.6), 179.0338 (5.5), 161.0231 (2.0), 147.0285 (3.9), 135.0437 (8.0), 129.0179 (10.8), 111.0072 (2.1), 85.0279 (45.0)	6.44	1.133	2	
78.	tricaffeoylhexaric acid[b] 4	$C_{33}H_{28}O_{17}$	695.1254	695.1268 (19.2), 533.0936 (51.1), 371.0621 (24.2), 209.0295 (100), 191.0188 (17.8), 179.0339 (5.4), 173.0448 (2.5), 161.0230 (4.1), 147.0285 (4.4), 135.0437 (10.6), 129.0179 (7.7), 85.0279 (45.1)	6.53	2.011	2	
79.	hydroxybutanyl-trcaffeoylhexaric acid[b] 1	$C_{37}H_{34}O_{19}$	781.1622	781.1623 (34.0), 619.1307 (44.6), 457.0989 (38.4), 353.0515 (0.8), 295.0670 (52.9), 209.0291 (2.9), 191.0187 (78.8), 179.0338 (10.9), 173.0077 (5.3), 161.0232 (6.6), 147.0285 (16.4), 135.0437 (19.0), 129.0178 (17.4), 111.0073 (5.4), 85.0278 (100)	6.53	0.177	2	
80.	hydroxybutanyl-trcaffeoylhexaric acid[b] 2	$C_{37}H_{34}O_{19}$	781.1622	781.1634 (42.3), 619.1310 (54.7), 457.0990 (36.0), 353.0516 (0.6), 295.0670 (65.8), 209.0289 (0.8), 191.0187 (66.9), 179.0337 (9.7), 173.0079 (4.1), 161.0234 (7.3), 147.0286 (16.6), 135.0437 (19.1), 129.0179 (17.9), 111.0072 (6.4), 85.0279 (100)	6.66	1.585	2	
81.	isobutanyl-tricaffeoylhexaric acid[b]	$C_{37}H_{34}O_{18}$	765.1672	765.1686 (59.7), 603.1352 (50.7), 441.1042 (35.0), 279.0720 (92.3), 191.0188 (68.4), 179.0337 (13.7), 173.0078 (4.4), 161.0231 (8.1), 147.0288 (10.5), 135.0437 (25.3), 129.0179 (14.2), 111.0073 (6.5), 85.0278 (100)	8.54	1.755	2	

82.	2-methyllbutanyl/ isovaleryl-tricaffeoylhexaric acid[b]	$C_{38}H_{36}O_{18}$	779.1829	779.1841 (49.7), 617.1520 (41.8), 455.1198 (28.0), 293.0880 (76.2), 191.0187 (62.2), 179.0330 (15.1), 173.0079 (5.5), 161.0233 (6.7), 147.0286 (12.3), 135.0437 (27.8), 129.0179 (0.7), 111.0070 (7.1), 85.0278 (100)	9.21	1.531	2	
				**Flavonoids**				
83.	myricetin *O*-hexoside	$C_{21}H_{20}O_{13}$	479.0831	479.0832 (77.0), 359.0386 (0.4), 317.0301 (100), 287.0189 (2.0), 271.0252 (3.1), 243.0300 (1.9), 227.0343 (1.2), 215.0343 (0.4), 199.0387 (1.1), 178.9977 (2.0), 151.0025 (0.8)	4.55	0.159	2	[1, 2]
84.	myricetin *O*-acetylhexoside	$C_{23}H_{22}O_{14}$	521.0939 (	521.0939 (76.9), 461.0714 (0.2), 317.0301 (100), 287.0197 (2.2), 271.0249 (3.5), 243.0295 (2.2), 227.0341 (1.5),	5.19	0.483	2	[1]
85.	hyperoside[a]	$C_{21}H_{20}O_{12}$	463.0885	463.0884 (100), 301.0348 (44.3), 300.0275 (71.3), 271.0247 (37.4), 255.0296 (16.5), 243.0296 (8.6), 227.0340 (2.8), 178.9973 (2.6), 163.0024 (2.2), 151.0023 (6.0), 121.0275 (1.5), 107.0121 (1.8)	5.29	0.520	1	[1, 2]
86.	luteolin-7-*O*-glucoside[a,b]	$C_{21}H_{20}O_{11}$	447.0934	447.0934 (100), 285.0401 (39.5), 284.0326 (57.5), 255.0299 (0.8), 239.0346 (0.6), 227.0340 (3.2), 211.0392 (2.4)	5.31	0.213	1	
87.	patuletin *O*-hexoside[b]	$C_{22}H_{22}O_{13}$	493.0987	493.0992 (100), 331.0460 (78.6), 316.0222 (17.3), 287.0199 (20.4), 271.0246 (7.6), 259.0250 (4.0), 243.0303 (2.9), 215.0340 (2.3), 199.03962 (1.2), 181.0132 (4.5), 165.9895 (5.1), 139.0020 (2.3), 136.9866 (2.6), 121.0280 (3.7)	5.50	0.783	2	
88.	quercetin *O*-acetylhexoside[b] 1	$C_{23}H_{22}O_{13}$	505.0988	505.0991 (100), 463.0863 (1.1), 301.0345 (33.8), 300.0275 (79.9), 271.0247 (34.5), 255.0296 (15.0), 243.0295 (9.23), 227.0346 (2.5), 211.0396 (0.8), 199.0392 (0.5), 178.9968 (2.5), 163.0026 (1.7), 151.0024 (4.8), 135.0070 (0.5), 121.0270 (0.9), 107.0123 (1.8)	5.61	0.666	2	
89.	nepetin *O*-hexoside[b]	$C_{22}H_{22}O_{12}$	447.1038	477.1038 (100), 357.0618 (0.6), 315.0465 (26.0), 300.0271 (15.1), 299.0197 (18.6), 271.0251 (2.9), 255.0299 (1.4), 243.0296 (2.9), 227.0343 (2.8), 165.9898 (0.6), 163.0024 (1.8), 164.9816 (1.6), 136.9867 (1.3), 133.0280 (12.2)	5.68	-0.061	2	
90.	kaempferol 3-*O*-glucoside[a]	$C_{21}H_{20}O_{11}$	447.0934	447.0934 (100), 285.0396 (23.9), 284.0326 (55.8), 255.0296 (44.0), 227.0344 (42.4), 211.0397 (1.5), 178.9980 (0.4), 163.0022 (0.4), 151.0023 (2.2), 107.0120 (0.9)	5.88	0.258	1	[1, 2]

91.	isorhamnetin 3-*O*-glucoside[a]	$C_{22}H_{22}O_{12}$	477.1038	477.1038 (100), 357.0618 (1.9), 315.0496 (12.6), 314.0434 (54.8), 299.0197 (4.4), 271.0248 (20.3), 257.0456 (4.5), 243.0293 (23.6), 227.0340 (3.0), 215.0349 (3.2), 199.0391 (3.0), 151.0020 (2.6)	6.03	-0.124	1	[1, 2]
92.	quercetin *O*-acetylhexoside isomer[b] 2	$C_{23}H_{22}O_{13}$	505.0988	505.0983 (100), 301.0352 (97.8), 271.0247 (2.4), 255.0292 (1.9), 243.0298 (1.3), 227.0339 (1.6), 211.0392 (0.7), 161.0232 (4.8), 151.0023 (35.2), 121.0280 (8.2), 107.0123 (14.5)	6.06	-0.859	2	
93.	quercetin O-malonylhexoside	$C_{24}H_{22}O_{15}$	549.0886	549.0860 (0.7), 505.0995 (100), 463.0898 (0.5), 343.0456 (2.8), 313.0360 (2.3), 301.0354 (90.3), 271.0247 (1.2), 255.0295 (1.0), 243.0303 (0.5), 227.0342 (0.8), 178.9978 (1.9), 175.0023 (4.2), 151.0023 (32.2), 121.0280 (7.1), 107.0123 (13.3)	6.06	-4.722	2	[1]
94.	6-methoxykaempferol *O*-hexoside[b]	$C_{22}H_{22}O_{12}$	477.1038	477.1032 (100), 357.0626 (0.8), 314.0434 (39.7), 315.0508 (29.9), 299.0198 (19.6), 271.0247 (13.3), 255.0295 (1.6), 243.0295 (4.1), 227.0346 (2.5), 215.0343 (6.9), 199.0393 (1.5), 181.0132 (5.2), 165.9896 (14.2), 109.9993 (4.2)	6.25	-1.340	2	
95.	kaempferol *O*-acetylhexoside	$C_{23}H_{22}O_{12}$	489.1038	489.1040 (100), 285.0401 (73.9), 255.0296 (45.1), 227.0344 (29.6), 211.0392 (2.8), 199.0388 (0.6), 163.0025 (1.3), 151.0020 (1.6)135.0070 (1.5), 107.0122 (2.0)	6.28	0.308	2	[1]
96.	hispidulin *O*-hexoside[b]	$C_{22}H_{22}O_{11}$	461.1089	461.1093 (100), 299.0555 (8.1), 284.0313 (9.2), 283.0247 (43.5), 255.0297 (10.0), 211.0396 (1.5), 183.0441 (3.2), 163.0023 (10.1), 135.0073 (2.3), 117.0330 (7.5)	6.32	0.792	2	
97.	jaceosidine *O*-hexoside[b]	$C_{23}H_{24}O_{12}$	491.1195	491.1197 (100), 329.0664 (4.6), 314.0421 (8.6), 313.0355 (11.8), 298.0118 (11.8), 270.0167 (13.2), 257.0074 (0.9), 242.0217 (2.4), 214.0268 (1.6), 198.0312 (1.2), 165.9886 (0.4), 164.9817 (1.1), 163.0022 (2.1), 147.0437 (2.0), 136.9867 (2.0), 132.0200 (1.8)	6.50	1.875	2	
98.	quercetin *O*-coumaroylhexoside isomer	$C_{30}H_{26}O_{14}$	609.1250	609.1254 (100), 463.0888 (23.6), 301.0350 (42.5), 300.0276 (60.9), 271.0248 (36.9), 255.0297 (17.6), 243.0294 (9.1), 227.0344 (2.7), 211.0400 (0.7), 199.0392 (0.6), 178.9976 (2.7), 163.0023 (1.9), 151.0023 (7.9), 121.0280 (1.5), 107.0122 (2.9)	7.07	0.643	2	[1, 2]
99.	quercetin *O*-feruloylhexoside[b]	$C_{31}H_{28}O_{15}$	639.1355	639.1357 (100), 463.0883 (11.5), 301.0349 (29.9), 300.0276 (55.5), 271.0248 (34.5), 255.0298 (16.3), 243.0297 (9.2), 227.0342 (2.8), 211.0397 (0.9), 199.0398	7.20	0.308	2	

				(1.8), 161.0231 (9.6), 151.0024 (7.9), 135.0438 (7.9), 121.0283 (1.3), 107.0122 (2.8)				
100.	quercetin *O*-coumaroylhexoside 2	$C_{30}H_{26}O_{14}$	609.1250	609.1254 (100), 463.0885 (26.0), 301.0350 (51.3), 300.0275 (78.1), 283.0245 (0.3), 271.0248 (43.7), 255.0298 (19.1), 243.0295 (10.5), 227.0345 (2.6), 211.0392 (1.4), 199.0394 (0.5), 178.9975 (3.6), 163.0025 (2.2), 151.0023 (9.3), 121.0281 (1.9), 107.0123 (3.2)	7.30	0.643	2	[1, 2]
101.	luteolin[a,b]	$C_{15}H_9O_7$	285.0406	285.0402 (100), 267.0292 (0.2), 257.0443 (0.2), 241.0499 (0.8), 223.0388 (0.2), 217.0499 (0.9), 199.0394 (1.7), 175.0390 (2.8), 151.0024 (4.4), 133.0280 (21.6), 107.0123 (4.1)	7.58	-0.952	1	
102.	quercetin[a]	$C_{15}H_{10}O_7$	301.0354	301.0353 (100), 273.0406 (3.2), 257.0459 (1.1), 243.0297 (0.3), 229.6005 (0.7), 211.0389 (0.3), 178.9975 (21.6), 151.0023 (47.4), 175.0391 (0.3), 121.0280 (12.3), 107.0123 (12.9)	7.62	-0.252	1	[2]
103.	kaempferol *O*-coumaroylhexoside 1	$C_{30}H_{26}O_{13}$	593.1301	593.1307 (100), 447.0934 (2.7), 285.0401 (75.2), 255.0297 (42.7), 227.0343 (28.3), 211.0391 (2.5), 163.0388 (1.3), 151.0024 (3.2), 107.0121 (2.5)	7.69	1.039	2	[1, 2]
104.	patuletin[b]	$C_{16}H_{12}O_8$	331.0464	331.0458 (100), 316.0223 (62.3), 287.0198 (12.6), 271.0245 (7.0), 259.0246 (3.7), 243.0303 (1.2), 242.0211 (1.2), 181.0129 (5.2), 165.9895 (18.3), 139.0023 (9.5), 136.9858 (0.6), 121.0280 (1.2), 109.9994 (11.5)	7.72	-0.304	2	
105.	herbacetin methyl ether	$C_{16}H_{12}O_7$	315.0510	315.0512 (82.8), 300.0276 (100), 271.0251 (0.4), 255.0295 (1.7), 243.0290 (2.0), 227.0346 (2.3)	7.76	0.679	2	[1]
106.	nepetin[b]	$C_{16}H_{12}O_7$	315.0510	315.0512 (82.8), 300.0276 (100), 271.0251 (1.2), 255.0295 (1.7), 243.0290 (2.0), 227.0346 (2.3), 201.0184 (3.7), 165.9897 (1.2), 136.9866 (9.7), 109.9904 (1.2)	7.76	0.679	2	
107.	isorhamnetin *O*-*p*-coumaroylhexoside isomer[b] 1	$C_{31}H_{28}O_{14}$	623.1406	623.1412 (100), 477.1039 (1.4), 315.0511 (69.2), 299.0197 (15.7), 300.0271 (10.4), 271.0248 (24.4), 255.0298 (8.9), 243.0295 (14.4), 227.0345 (4.0), 199.0395 (3.6), 151.0023 (5.0), 145.0281 (10.0), 107.0125 (1.3)	7.88	0.981	2	
108.	kaempferol *O*-coumaroylhexoside isomer 2	$C_{30}H_{26}O_{13}$	593.1301	593.1307 (100), 447.0934 (1.7), 285.0403 (69.5), 255.0296 (34.2), 227.0343 (25.2), 211.0394 (2.2), 161.0232 (2.0), 151.0021 (2.6), 135.0430 (0.7), 107.0122 (2.3)	7.95	1.072	2	[1, 2]
109.	kaempferol *O*-caffeoylhexoside[b]	$C_{30}H_{26}O_{14}$	609.1250	609.1252 (100), 429.0809 (0.5), 447.0910 (0.8), 285.0404 (59.1), 255.0295 (10.2), 227.0344 (9.7), 179.0339 (24.8), 161.0232 (48.9), 135.0437 (28.8), 151.0023 (3.2), 107.0123 (1.6)	7.99	0.364	2	

110.	isorhamnetin *O-p*-coumaroylhexoside isomer[b] 2	$C_{31}H_{28}O_{14}$	623.1406	623.1414 (100), 477.1046 (1.5), 315.0510 (74.0), 299.0197 (17.7), 300.0273 (15.6), 271.0248 (30.1), 255.0298 (10.5), 243.0290 (17.4), 227.0345 (4.6), 199.0393 (5.2), 163.0388 (2.6), 151.0023 (5.3), 145.0281 (11.7), 107.0122 (1.6)	8.08	1.174	2	
111.	isorhamnetin *O*-caffeoylhexoside[b]	$C_{31}H_{28}O_{15}$	639.1355	639.1359 (100), 477.1042 (2.0), 315.0512 (68.3), 301.0312 (0.9), 300.0280 (13.4), 271.0247 (17.4), 255.0290 (5.7), 243.0295 (7.9), 227.0347 (1.6), 199.0386 (0.8), 179.0339 (25.8), 161.0232 (70.4), 135.0437 (36.3), 107.0121 (0.9)	8.12	0.496	2	
112.	axillarin[b] (quercetagetin 3,6-dimethylether)	$C_{17}H_{14}O_8$	345.0616	345.0616 (100), 330.0382 (81.6), 315.0148 (69.7), 287.0200 (11.6), 259.0252 (3.6), 243.0298 (3.5), 231.0297 (5.3), 215.0348 (4.2), 175.0026 (3.4), 165.9892 (6.1), 149.0230 (11.2), 139.0387 (5.4), 136.9865 (0.7), 109.9994 (4.8)	8.19	0.027	2	
113.	hispidulin[b]	$C_{16}H_{12}O_6$	299.0561	299.0558 (62.0), 284.0324 (100), 256.0374 (0.8), 255.0310 (16), 227.0346 (13.1), 212.0471 (2.5), 165.9896 (0.9), 136.9866 (13.2)	8.84	0.430	2	
114.	kaempferol[a]	$C_{15}H_9O_7$	285.0406	285.0403 (100), 257.0458 (0.8), 239.0346 (1.3), 229.0446 (0.8), 211.0396 (1.0), 151.0027 (1.5), 117.0329 (1.0), 107.0127 (1.2)	8.84	-0.531	1	[2]
115.	naringenin	$C_{15}H_{12}O_5$	271.0612	271.0611 (100), 253.0504 (6.8), 243.0656 (0.7), 225.0558 (1.8), 215.0708 (1.0), 197.0600 (5.0), 151.0022 (3.4), 119.0490 (0.3), 107.0122 (2.8)	8.86	0.468	1	[1]
116.	isorhamnetin[a]	$C_{16}H_{12}O_7$	315.0510	315.0510 (100), 300.0275 (45.7), 271.0246 (2.9), 255.0293 (1.5), 243.0296 (1.5), 227.0356 (1.2), 215.0344 (0.7), 164.0103 (3.1), 151.0024 (9.7), 107.0123 (7.7)	9.12	0.013	1	[1, 2]
117.	jaceosidin[b] (6-hydroxyluteolin-6,3'-dimethyl ether)[a,b]	$C_{17}H_{14}O_7$	329.0677	329.0668 (83.8), 314.0434 (97.2), 299.0197 (100), 271.0247 (22.1), 255.0287 (0.8), 243.0295 (6.4), 227.0345 (4.0), 215.0341 (17.8), 199.0394 (4.8), 187.0387 (0.6), 165.9895 (16.2), 164.9815 (6.3), 136.9862 (2.0), 133.0278 (9.1)	9.40	0.286	1	
118.	gnaphaliin 1	$C_{17}H_{14}O_6$	313.0718	313.0719 (94.4), 298.0482 (100), 283.0248 (95.5), 255.0296 (52.8), 239.0345 (1.5), 227.0341 (6.3), 211.0391 (3.5), 199.0392 (18.0), 183.0441 (13.1), 167.0488 (7.6), 155.0487 (6.8), 139.0538 (6.9), 127.0539 (2.2), 109.0644 (3.1)	9.52	0.443	2	[1, 2]
119.	quercetagetin-3,6,3'(4')-trimethyl ether[b] 1	$C_{18}H_{16}O_8$	359.0772	359.0774 (100), 344.0538 (59.7), 329.0302 (80.3), 314.0070 (2.6), 301.0354 (7.6), 286.0120 (20.3), 258.0168 (15.4), 230.0213 (8.2), 202.0263 (9.0),	9.66	0.444	2	

				165.9895 (2.2), 163.0380 (2.1), 136.9870 (0.5), 118.0155 (1.1), 109.9997 (2.2)				
120.	quercetin 7,3'(4')-dimethyl ether[b]	$C_{17}H_{13}O_7$	329.0667	329.0667 (100), 314.0432 (81.6), 299.0196 (88.2), 271.0247 (33.5), 255.0292 (0.8), 243.0295 (12.5), 227.0340 (3.0), 215.0340 (4.9), 199.0393 (13.9), 171.0439 (143.0489 (3.1), 155.0486 (6.5)	9.71	0.073	2	
121.	kaempferid/isokampferid	$C_{16}H_{12}O_6$	299.0561	299.0561 (100), 284.0327 (77.7), 255.0296 (55.5), 239.0346 (43.4), 227.0344 (26.1), 211.0394 (14.5), 199.0388 (0.5), 109.9995 (0.3)	9.99	-0.561	2	[1]
122.	quercetagetin-3,6,3'(4')-trimethyl ether[b] 2	$C_{18}H_{16}O_8$	359.0772	359.0772 (100), 344.0535 (30.5), 329.0302 (48.1), 314.0082 (2.4), 301.0359 (3.1), 286.0120 (8.6), 258.0175 (1.5), 230.0215 (3.7), 194.9925 (25.2), 179.9690 (18.7)	11.18	-0.002	2	
123.	pinocembrin	$C_{15}H_{12}O_4$	255.0663	255.0659 (100), 227.0703 (0.3), 211.0758 (1.8), 183.0808 (0.3), 151.0024 (5.4), 107.0123 (5.9)	11.63	-1.302	2	[1, 2]
124.	galangin	$C_{15}H_{10}O_5$	269.0457	269.0453 (100), 239.0347 (0.6), 227.0348 (0.7), 223.0402 (1.0), 213.0552 (1.5), 195.0446 (1.1), 185.0592 (0.5), 169.0647 (1.9), 143.0489 (1.3), 107.0121 (0.2), 101.0379 (0.2)	11.74	-0.954	2	[2]
125.	gnaphaliin 2	$C_{17}H_{14}O_6$	313.0718	313.0718 (100), 298.0481 (88.6), 283.0246 (74.6), 255.0296 (0.4), 239.0343 (0.5), 227.0343 (4.4), 211.0387 (2.8), 199.0392 (12.6), 183.0441 (7.9), 167.0492 (5.2), 155.0486 (4.7), 139.0538 (4.7), 127.0538 (2.4), 117.0328 (0.3)	12.19	0.123	2	[1, 2]
126.	galangin methyl ether	$C_{16}H_{12}O_5$	283.0612	283.0611 (100), 268.0376 (77.3), 239.0346 (54.3), 211.0393 (76.2), 195.0439 (2.0), 167.0485 (2.3)	12.27	-0.342	2	[1]

Pyrones (phloroglucinol alpha-pyrones)

**№**	**Subclass (tentatively identified compound)**	**Molecular formula**	**Exact mass [M-H]⁻**	**Fragmentation pattern in (-) ESI-MS/MS**	**t_R (min)**	**Δ ppm**	**Level of confidence**	**References**
127.	ethylpyrones A[b,c]	$C_{22}H_{26}O_8$	417.1555	417.1557 (42.8), 263.0929 (3.2), 251.0922 (100), 233.0815 (1.4), 205.0496 (0.4), 193.0496 (18.1), 179.0339 (3.1), 153.0544 (73.5), 109.0643 (49.0)	13.15	0.429	3	
128.	ethylpyrones[b,c]	$C_{22}H_{26}O_9$	433.1504	433.1502 (66.6), 378.0953 (4.1), 346.1021 (0.7), 279.0871 (33.1), 267.0872 (100), 261.0765 (1.9), 249.0764 (2.8), 235.0970 (32.1), 223.0969 (49.7), 193.0496 (0.4), 179.0338 (0.4), 179.1065 (17.6), 151.0387 (9.5), 125.0592 (11.0), 109.0643 (4.7)	13.46	-0.475	3	
129.	ethylpyrones[b,c]	$C_{24}H_{28}O_8$	443.1711	443.1710 (100), 289.1081 (81.5), 277.1079 (74.3), 271.0970 (1.6), 259.0973 (19.0), 247.0974 (2.3),	14.15	-0.341	3	

				235.0974 (2.4), 191.0341 (1.7), 179.0336 (6.4), 167.0337 (18.4), 153.0544 (7.5), 123.0435 (7.6), 109.0643 (11.5)				
130.	isopropylpyrones[b,c]	$C_{23}H_{28}O_9$	447.1661	447.1660 (76.3), 279.00872 (28.8), 267.0872 (100), 253.0715 (8.8), 235.0970 (33.6), 223.0970 (56.7), 209.0807 (4.0), 179.1066 (17.3), 167.0703 (2.0), 151.0386 (10.5), 125.0594 (11.0), 97.0278 (3.4)	14.52	-0.147	3	
131.	methylpyrones (arenol)	$C_{21}H_{24}O_7$	387.1449	387.1450 (64.2), 247.0972 (85.5), 235.0970 (100), 205.0860 (6.3), 191.1068 (11.3), 187.0753 (0.7), 166.0262 (4.6), 161.0959 (2.9), 133.0643 (0.8), 123.0435 (2.7), 109.0640 (5.1), 95.0486 (16.1)	14.65	0.087	2	[3]
132.	ethylpyrones[b,c]	$C_{27}H_{34}O_8$	485.2181	485.2183 (100), 331.1552 (57.5), 319.1550 (72.4), 289.1454 (0.4), 277.1455 (1.5), 258.1167 (0.3), 191.0340 (6.2), 179.0340 (10.4), 166.0261 (3.9), 153.0544 (8.7), 137.0229 (3.6), 109.0641 (11.3)	14.89	0.472	3	
133.	unknown[b,c]	$C_{20}H_{26}O_6$	361.1657	361.1660 (95.7), 317.1757 (100), 273.1859 (8.2), 205.0500 (6.2), 193.0497 (22.3), 165.0542 (0.5), 163.0382 (3.9), 151.0388 (6.2), 123.0431 (1.7), 107.0484 (0.9)	15.07	0.937	3	
134.	ethylpyrones [b,c]	$C_{23}H_{28}O_9$	447.1661	447.1659 (100), 293.1031 (61.8), 281.1029 (55.9), 261.0767 (23.3), 249.0765 (49.7), 233.0814 (23.5), 221.0812 (35.1), 153.0544 (7.7), 137.0230 (7.8), 109.0643 (8.0)	15.23	-0.370	3	
135.	ethylpyrones (arzanol)	$C_{22}H_{26}O_7$	401.1606	401.1605 (75.7), 247.0972 (82.9), 235.0970 (100), 219.1019 (0.8), 205.0857 (5.5), 191.1067 (13.4), 187.0760 (0.6), 166.0257 (6.6), 153.0543 (15.1), 123.0436 (1.2), 109.0641 (18.1)	15.76	-0.140	2	[3]
136.	ethylpyrones [b,c]	$C_{27}H_{34}O_8$	485.2181	485.2181 (100), 331.1552 (75.7), 319.1550 (97.9), 277.1438 (3.0), 205.0498 (11.6), 191.0334 (3.2), 179.0335 (2.1), 166.0260 (7.5), 153.0543 (8.8), 123.0437 (4.3), 109.0643 (10.2)	15.81	0.101	3	
137.	ethylpyrones [b,c]	$C_{24}H_{30}O_9$	461.1817	461.1796 (11.4), 307.1194 (13.6), 295.1187 (100), 251.1286 (3.6), 207.0656 (3.4), 153.0544 (32.4), 109.0643 (45.5)	16.30	0.012	3	
138.	ethylpyrones B[b,c]	$C_{25}H_{32}O_8$	459.2024	459.2023 (63.4), 305.1397 (6.7), 293.1394 (100), 279.1239 (4.2), 247.0981 (2.6), 235.0971 (14.0), 221.0806 (2.9), 191.1066 (4.9), 153.0544 (68.2), 109.0643 (55.5)	17.01	-0.307	3	
139.	isopropylpyrones[b,C]	$C_{23}H_{28}O_7$	415.1751	415.1762 (76.3), 247.0972 (78.9), 235.0970 (100), 205.0869 (4.1), 191.1068 (8.9), 167.0701 (12.5), 123.0800 (16.7), 109.0643 (1.0)	17.05	0.033	3	

140.	ethylpyrones[b,c]	$C_{22}H_{26}O_8$	417.1555	417.1554 (87.9), 263.0923 (76.6), 251.0922 (100), 233.0814 (12.9), 217.0517 (0.4), 209.0815 (1.4), 193.0490 (4.2), 164.0466 (12.3), 179.0341 (3.8), 153.0542 (4.8), 109.0644 (6.6)	17.11	-0.146	3	
141.	ethylpyrones[b,c]	$C_{23}H_{28}O_7$	415.1751	415.1763 (91.5), 261.1131 (78.0), 249.1129 (100), 243.1026 (0.4), 233.1191 (0.4), 217.1221 (0.6), 205.1225 (10.0), 193.0859 (4.8), 180.0418 (3.3), 153.0545 (12.4), 109.0643 (16.4)	17.81	0.177(	3	
142.	ethylpyrone[b,c]	$C_{24}H_{28}O_8$	443.1711.	443.1709 (64.4), 289.1081 (21.4), 277.1079 (64.0), 235.0967 (1.8), 205.0862 (46.3), 193.0860 (100), 153.0543 (7.5), 109.0643 (14.4)	17.89	-0.476	2	
143.	1-methyl-propylpyrones[b,c]	$C_{24}H_{30}O_7$	429.1919	429.1917 (83.2), 247.0972 (77.5), 235.0970 (98.8), 205.0863 (4.9), 191.1069 (12.5), 181.0857 (13.4), 137.0958 (15.7), 150.0314 (3.2), 109.0642 (1.7)	18.55	-0.458	3	
144.	isopropylpyrone[b,c]	$C_{23}H_{28}O_8$	431.1711	431.1769 (95.2), 263.0923 (70.3), 251.0922 (100), 233.0815 (16.4), 209.0800 (1.2), 193.0496 (5.1), 179.0336 (4.8), 167.0701 (5.7), 164.0467 (16.4), 123.0800 (2.1), 109.0646 (0.4)	19.06	-0.489	3	
145.	unknown[b,c]	$C_{23}H_{32}O_6$	403.2126	403.2127 (100), 385.2027 (0.6), 359.2229 (62.3), 315.2324 (5.8), 247.0974 (2.3), 235.0972 (11.8), 217.0857 (1.6), 207.1016 (0.6), 191.0338 (1.5), 151.0388 (4.0), 125.0594 (6.4)	19.22	0.128	3	
146.	ethylpyrones[b] (6-*O*-desmethyl-auricepyrone)	$C_{24}H_{30}O_7$	429.1919	429.1917 (91.5), 275.1288 (82.2), 263.1286 (100), 257.1179 (0.9), 245.1179 (0.8), 229.1234 (0.8), 219.1383 (11.0), 193.0863 (3.5), 153.0543 (14.5), 109.0643 (24.2)	19.57	-0.458	2	[4, 5]
147.	ethylpyrones[b,c]	$C_{23}H_{28}O_8$	431.1711	431.1707 (100), 277.1079 (70.3), 265.1079 (90.6), 247.0973 (13.5), 221.1177 (0.7), 207.0647 (4.5), 193.0496 (4.2), 178.0624 (11.7), 153.0542 (6.9), 109.0642 (10.7),	19.86	-0.976	3	
148.	methylpyrones[b]	$C_{26}H_{32}O_7$	455.2075	455.2078 (100), 315.1602 (73.8), 303.1601 (88.1), 273.1501 (1.3), 259.1696 (4.3), 246.0892 (1.5), 231.0652 (3.1), 218.0939 (0.8), 179.0339 (12.2), 139.0386 (12.2)	20.19	0.513	3	[1, 6, 7]
149.	1-methyl-propylpyrones (heliarzanol/isobar)	$C_{24}H_{30}O_8$	445.1868	445.1865 (97.8), 263.0922 (67.0), 251.0922 (100), 245.0827 (0.5), 233.0813 (14.9), 209.0819 (1.0), 193.0494 (4.6), 181.0861 (6.0), 137.0958 (8.8), 109.0642 (1.5)	20.39	-0.744	3	
150.	1-methyl-propylpyrones[b,c]	$C_{24}H_{28}O_7$	427.1762	427.1737 (19.5), 245.0809 (0.8), 233.0814 (100), 189.0909 (7.5), 181.0859 (59.0), 137.0957 (43.2), 109.0648 (0.4)	20.42	-5.914	3	

№	Identified/tentatively annotated compound	Molecular formula	Exact mass	Fragmentation pattern in (+) ESI-MS/MS	t_R (min)	Δ ppm	Level of confidence	References
151.	ethylpyrones[b] (23-methyl-6-*O*-desmethylauricepyrone)	$C_{25}H_{32}O_7$	443.2075.	443.2075 (64.4), 289.1445 (78.4), 277.1442 (100), 233.1544 (8.6), 219.0656 (3.8), 205.0496 (4.6), 193.0860 (3.2), 153.0543 (14.0), 109.0643 (16.3	20.73	-0.060	3	[5, 8]
152.	ethylpyrones (heliarzanol/isobar)	$C_{24}H_{30}O_8$	445.1868	445.1867 (100), 291.1237 (61.3), 279.1235 (84.8), 273.1115 (0.6), 261.1133 (11.0), 221.0808 (6.0), 165.0907 (4.7), 153.0546 (5.3), 109.0642 (19.4)	21.00	-0.182	2	[1, 6]
153.	ethylpyrones[b]	$C_{27}H_{34}O_7$	469.2232	469.2232 (100), 315.1600 (49.9), 303.1601 (60.2), 273.1493 (1.0), 259.1696 (3.4), 245.0810 (1.0), 231.0660 (1.9), 191.0338 (5.8), 179.0340 (10.8), 166.0260 (12.6), 153.0546 (7.0), 109.0643 (8.7)	21.23	0.050	2	[7]
154.	ethylpyrones B[b,c]	$C_{25}H_{32}O_8$	459.2024	459.2026 (100), 305.1395 (58.8), 293.1395 (80.5), 275.1289 (9.7), 235.0976 (3.1), 219.0654 (13.9), 191.0704 (0.8), 179.1066 (3.9), 153.0542 (4.5), 109.0642 (8.8)	21.67	0.368	3	
155.	ethylpyrones[b,c]	$C_{27}H_{34}O_8$	485.2181	485.2181 (100), 331.1551 (50.4), 319.1549 (66.3), 301.1446 (6.1), 289.1440 (0.5), 277.1442 (1.3), 219.0649 (0.8), 193.0499 (2.8), 179.0338 (4.7), 153.0541 (4.5), 137.0593 (3.3), 109.0693 (3.7)	21.97	-0.023	3	
156.	Ethylpyrones C [b,c]	$C_{24}H_{28}O_7$	427.1762	427.1763 (22.3), 273.1131 (3.9), 261.1129 (100), 243.1017 (0.4), 217.1223 (4.0), 199.1114 (0.5), 191.0701 (1.6), 153.0543 (46.5), 109.0643 (43.1)	22.13	0.102	2	
157.	isopropylpyrones[b]	$C_{28}H_{36}O_7$	483.2388	483.2388 (89.2), 315.1600 (78.8), 303.1601 (100), 273.1496 (1.8), 259.1699 (4.5), 191.0341 (9.1), 179.0338 (16.9), 167.0701 (3.7), 166.0259 (5.1), 123.0801 (17.0)	22.41	-0.055	3	[8]
158.	Ethylpyrones C [b,c]	$C_{24}H_{28}O_7$	427.1762	427.1762 (40.7), 273.1133 (15.0), 261.1129 (100), 217.1226 (2.6), 191.0702 (18.9), 153.0543 (18.9), 109.0642 (15.9)	23.01	-0.038	2	
				**Other compounds**				
159.	gnaphaliol *O*-hexoside	$C_{19}H_{24}O_9$	395.1348	395.1323 (8.8), 377.1234 (0.3), 233.0814 (100), 189.0912 (8.1), 173.0595 (1.2), 147.0800 (4.0), 83.0121 (5.5)	8.14	-6.316	2	[1]
160.	micropyrone	$C_{14}H_{20}O_4$	251.1289	251.1286 (48.7), 207.0382 (100), 179.1064 (1.8), 151.1115 (30.0), 113.0956 (90.1), 85.0642 (28.4)	9.12	-1.045	2	[6]
161.	italipyrone 1	$C_{22}H_{24}O_7$	399.1449	399.1444 (11.8), 233.0814 (100), 189.0911 (7.1), 173.0594 (0.8), 153.0543 (61.3), 109.0643 (36.9)	17.33	-1.369	2	[1]
162.	Italipyrone 2	$C_{22}H_{24}O_7$	399.1449	399.1440 (14.7), 233.0813 (100), 189.0913 (4.7), 173.0597 (1.1), 153.0543 (49.2), 109.0643 (35.5)	20.39	-2.231	2	[1]

			[M+H]+					
163.	6-hydroxytremeton	$C_{13}H_{14}O_3$	219.1013	219.1013 (65.9), 201.0911 (14.8), 183.0804 (100), 165.0700 (17.6), 159.0804 (79.7), 157.646 (23.2), 144.0569 (27.6), 141.0697 (12.2), 131.0855 (9.2), 129.0702 (4.3), 115.0546 (7.2), 107.0494 (5.8), 91.0548 (12.7), 57.0343 (3.4)	7.21	-1.373	3	[9]
164.	2-isobutyryl-6-acetylprenylphloroglucinol	$C_{17}H_{22}O_6$	323.1481	323.1481 (22.5), 305.1378 (53.1), 287.1254 (0.5), 277.1429 (62.1), 267.0858 (24.9), 249.0754 (100), 221.0805 (34.7), 203.0701 (66.6), 185.0600 (3.5), 175.0750 (13.6), 161.0595 (10.4), 147.0807 (4.8), 135.0805 (8.6), 109.0284 (1.9), 91.0546 (1.5), 79.0547 (0.5)	13.16	-2.522	3	[10]
165.	2-isobutyryl-4-prenylphloroglucinol	$C_{15}H_{20}O_4$	265.1431	265.1431 (34.3), 209.0807 (100), 191.0701 (32.7), 173.0598 (2.91), 163.0752 (15.8), 149.0597 (4.6), 135.0803 (3.7), 121.0649 (9.2), 113.0600 (2.2), 107.0859 (1.6), 93.0704 (1.3), 79.0548 (1.3)	17.68	-1.379	3	[10]
166.	2-metylvaleryl-4-prenylphloroglucinol	$C_{17}H_{24}O_4$	293.1741	293.1741 (9.8), 237.1118 (100), 219.1013 (28.0), 201.0910 (1.8), 191.1066 (3.6), 177.0909 (0.4), 163.0752 (2.4), 143.0853 (1.0), 131.0856 (1.6), 121.0650 (0.9), 107.0859 (0.7), 93.0705 (1.2), 91.0545 (1.8), 79.0551 (0.2)	18.28	-2.066	3	[10]

[a] identified by comparison with an authentic standard; [b] reported for the first time in *H. italicum*; [c]-undescribed in the literature; A, B, C compounds labeled with the same capital letters share the same fragmentation patterns; Level of confidence: 1-compound identified by comparison to reference standard; 2-putatively annotated compound; 3- putatively characterized compound classes.

Table S2. Tentative structures of prenylated phloroglucinol α-pyrones (for numbers and fragmentation patterns, see Table S1).

alpha-pyrone phloroglucinol

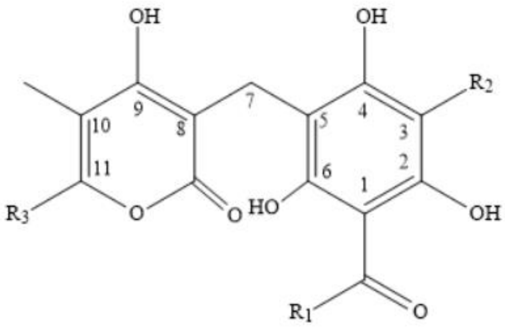

N	R_1	R_2	R3	$[M\text{-}H]^-$ Exact mass
		**Methylpyrones (MP)**		
**131**	CH_3	Prenyl(C_5H_9)	CH_3	387.1449
**148**	CH_3	Geranyl ($C_{10}H_{17}$)	CH_3	455.2075
		**Ethylpyrones (EP)**		
**135**	CH_3	Prenyl	$-CH_2-CH_3$	401.1606
**141**	$-CH_2-CH_3$	Prenyl	$-CH_2-CH_3$	415.1751
**146**	$-CH(CH_3)_2$	Prenyl	$-CH_2-CH_3$	429.1919
**151**	$CH(CH_3)-CH_2-CH_3$	Prenyl	$-CH_2-CH_3$	443.2075
**127**	CH_3	Hydroxyprenyl	$-CH_2-CH_3$	417.1555
**140**	CH_3	Hydroxyprenyl	$-CH_2-CH_3$	417.1555
**147**	$-CH_2-CH_3$	Hydroxyprenyl	$-CH_2-CH_3$	431.1711
**152**	$-CH(CH_3)_2$	Hydroxyprenyl	$-CH_2-CH_3$	445.1868
**138**	$-CH(CH_3)-CH_2-CH_3$	Hydroxyprenyl	$-CH_2-CH_3$	459.2024
**154**	$-CH(CH_3)-CH_2-CH_3$	Hydroxyprenyl	$-CH_2-CH_3$	459.2024
**153**	CH_3	Geranyl	$-CH_2-CH_3$	469.2232
**132**	CH_3	Hydroxygeranyl	$-CH_2-CH_3$	485.2181
**136**	CH_3	Hydroxygeranyl	$-CH_2-CH_3$	485.2181
**155**	CH_3	Hydroxygeranyl	$-CH_2-CH_3$	485.2181
**128**	CH_3	Dihydroxyprenyl	$-CH_2-CH_3$	433.1504
**134**	$-CH_2-CH_3$	Dihydroxyprenyl	$-CH_2-CH_3$	447.1661
**156**	$-CH(CH_3)_2$	$-C_5H_7$	$-CH_2-CH_3$	427.1762
**158**	$-CH(CH_3)_2$	$-C_5H_7$	$-CH_2-CH_3$	427.1762
**142**	$-C_3H_5OH$	Prenyl	$-CH_2-CH_3$	443.1711
		**Isopropylpyrones(IPP)**		
**139**	CH_3	Prenyl	$-CH(CH_3)_2$	415.1751
**157**	CH_3	Geranyl	$-CH(CH_3)_2$	483.2388
**144**	CH_3	Hydroxyprenyl	$-CH(CH_3)_2$	431.1711

**130**	CH_3	Dihydroxyprenyl	$-CH(CH_3)_2$	447.1661
		**1-Methyl-propylpyrones (MPP)**		
**150**	CH_3	C_5H_7	$-CH(CH_3)-CH_2-CH_3$	427.1762
**149**	CH_3	Hydroxyprenyl	$-CH(CH_3)-CH_2-CH_3$	445.1868
**143**	CH_3	Prenyl	$-CH(CH_3)-CH_2-CH_3$	429.1919

References

1. Kramberger, K.; Barlič-Maganja, D.; Bandelj, D.; Baruca Arbeiter, A.; Peeters, K.; Miklavčič Višnjevec, A.; Jenko Pražnikar, Z., HPLC-DAD-ESI-QTOF-MS determination of bioactive compounds and antioxidant activity comparison of the hydroalcoholic and water extracts from two Helichrysum italicum species. *Metabolites* **2020,** 10, (10), 403.
2. Pereira, C. G.; Barreira, L.; Bijttebier, S.; Pieters, L.; Neves, V.; Rodrigues, M. J.; Rivas, R.; Varela, J.; Custodio, L., Chemical profiling of infusions and decoctions of Helichrysum italicum subsp. picardii by UHPLC-PDA-MS and in vitro biological activities comparatively with green tea (Camellia sinensis) and rooibos tisane (Aspalathus linearis). *Journal of Pharmaceutical and biomedical analysis* **2017,** 145, 593-603.
3. Appendino, G.; Ottino, M.; Marquez, N.; Bianchi, F.; Giana, A.; Ballero, M.; Sterner, O.; Fiebich, B. L.; Munoz, E., Arzanol, an anti-inflammatory and anti-HIV-1 phloroglucinol α-pyrone from Helichrysum italicum ssp. microphyllum. *Journal of natural products* **2007,** 70, (4), 608-612.
4. Akaberi, M.; Sahebkar, A.; Azizi, N.; Emami, S. A., Everlasting flowers: Phytochemistry and pharmacology of the genus Helichrysum. *Industrial Crops and Products* **2019,** 138, 111471.
5. Jakupovic, J.; Kuhnke, J.; Schuster, A.; Metwally, M.; Bohlmann, F., Phloroglucinol derivatives and other constituents from South African Helichrysum species. *Phytochemistry* **1986,** 25, (5), 1133-1142.
6. Taglialatela-Scafati, O.; Pollastro, F.; Chianese, G.; Minassi, A.; Gibbons, S.; Arunotayanun, W.; Mabebie, B.; Ballero, M.; Appendino, G., Antimicrobial phenolics and unusual glycerides from Helichrysum italicum subsp. microphyllum. *Journal of natural products* **2013,** 76, (3), 346-353.
7. Tomás-Lorente, F.; Iniesta-Sanmartín, E.; Tomás-Barberán, F. A.; Trowitzsch-Kienast, W.; Wray, V., Antifungal phloroglucinol derivatives and lipophilic flavonoids from Helichrysum decumbens. *Phytochemistry* **1989,** 28, (6), 1613-1615.
8. Akaberi, M.; Danton, O.; Tayarani-Najaran, Z.; Asili, J.; Iranshahi, M.; Emami, S. A.; Hamburger, M., HPLC-based activity profiling for antiprotozoal compounds in the endemic Iranian medicinal plant Helichrysum oocephalum. *Journal of natural products* **2019,** 82, (4), 958-969.
9. Bohlmann, F.; Hoffmann, E., Cannabigerol-ähnliche verbindungen aus Helichrysum umbraculigerum. *Phytochemistry* **1979,** 18, (8), 1371-1374.

10. Lourens, A.; Viljoen, A. M.; Van Heerden, F., South African Helichrysum species: a review of the traditional uses, biological activity and phytochemistry. *Journal of Ethnopharmacology* **2008,** 119, (3), 630-652.
